# Semiochemicals and odorant receptors underlying potato cultivar susceptibility and resistance to potato tuber moth

**DOI:** 10.1073/pnas.2537754123

**Published:** 2026-04-06

**Authors:** Ruipeng Chen, Wangtao Hu, Qin Hu, Junjie Yan, Jiao Yin, Fathiya M. Khamis, Yulin Gao, Walter S. Leal

**Affiliations:** ^a^State Key Laboratory for Biology of Plant Diseases and Insect Pests, Institute of Plant Protection, Chinese Academy of Agricultural Sciences, Beijing 100193, China; ^b^International Center of Insect Physiology and Ecology, Nairobi 30772-00100, Kenya; ^c^Department of Zoology and Entomology, University of Pretoria, Hatfield 0028, South Africa; ^d^Institute of Bast Fiber Crops and Center of Southern Economic Crops, Chinese Academy of Agricultural Sciences, Changsha 410205, China; ^e^Department of Molecular and Cellular Biology, University of California, Davis, CA 95616

**Keywords:** nerolidol, carene, PopeOR01, PopeOR73, attractants and repellents

## Abstract

Infestation of potato by the potato tuber moth (*Phthorimaea operculella*) varies markedly among cultivars, yet the chemical and molecular mechanisms underlying this variation remain poorly understood. Here, we combine field surveys, chemical ecology, functional genomics, and structural modeling to reveal how cultivar-specific volatile profiles shape moth oviposition behavior and pest outcomes. Field and laboratory assays identified marked differences in adult attraction, oviposition, and larval damage among potato cultivars, which correlated with distinct blends of emitted volatile organic compounds (VOCs). Behavioral analyses revealed that among other VOCs, *trans*-nerolidol and β-ionone act as attractants, whereas 3-carene and benzyl tiglate function as oviposition deterrents. Electrophysiological screening and heterologous expression deorphanized six female-biased odorant receptors (ORs), identifying three narrowly tuned receptors linked to cultivar susceptibility or resistance. PopeOR01, upregulated following mating, selectively detects the attractant *trans*-nerolidol, which is enriched in susceptible cultivars, whereas PopeOR15 and PopeOR73 are tuned to repellents benzyl tiglate and 3-carene, respectively, which are enriched in resistant cultivars. CRISPR/Cas9-mediated knockouts confirmed that PopeOR01 and PopeOR73 are essential for antennal detection and behavioral responses to their cognate ligands. AlphaFold3 modeling, molecular docking, and molecular dynamics simulations revealed conserved OR-Orco channel architecture and stable ligand-binding modes consistent with physiological and behavioral data. Together, our results establish a mechanistic link between potato cultivar chemistry, insect olfactory receptor function, and pest behavior, providing a molecular framework for sustainable, semiochemical-based management of the potato tuber moth.

## Significance

Insect pests cause substantial losses in global food production, and their control often relies on chemical insecticides with environmental costs. Environmentally friendly alternatives, such as push–pull strategies that exploit plant-derived attractants and repellents, offer promising solutions but require a mechanistic understanding of insect–plant interactions. Here, we investigate the chemical ecology of the potato tuber moth, a major worldwide pest of potato. Through integrated field surveys, laboratory assays, and molecular analyses, we identify cultivar-emitted attractants and repellents and the olfactory receptors in gravid females that mediate host selection. These findings provide a foundation for cultivar-based and semiochemical-driven strategies to sustainably manage potato tuber moth populations.

Worldwide, plant pests and diseases reduce a substantial share of potential food production, leading to major economic losses and widespread reliance on insecticides for pest control (1, 2). To mitigate the environmental and health burdens associated with intensive insecticide use, a range of ecologically based pest-management strategies have been developed, including the push–pull approach (1). This strategy combines plant-derived repellents that “push” insects away from crops with attractive plants or traps that “pull” them toward designated areas. Push–pull systems have been successfully implemented in maize to control stem borers and fall armyworm (3–5), and in horticultural crops to manage pests such as onion thrips (6) and tephritid (Tephritidae) fruit flies (7). In southern Vietnam’s Mekong Delta, farmers have interplanted guava (*Psidium guajava*) among citrus (e.g., King mandarin) as part of an integrated pest management program targeting the Asian citrus psyllid (ACP, *Diaphorina citri*) and huanglongbing (HLB). Field observations indicate that citrus orchards interplanted with guava experience markedly lower psyllid infestations and reduced HLB incidence compared with citrus monocultures (8). Drawing on local ecological knowledge, farmers selected guava without knowing that it emits the sesquiterpene β-caryophyllene, a compound with repellent activity against ACP (9). Subsequent work demonstrating that Valencia sweet orange engineered to constitutively emit β-caryophyllene reduces ACP attraction (10) highlighted the potential for developing push–pull strategies using citrus varieties themselves.

A central component of push–pull strategies is the manipulation of host selection by gravid females. According to the preference–performance hypothesis (11, 12), gravid females maximize offspring fitness and minimize mortality risk by selecting oviposition sites that provide superior nutrition and reduced exposure to natural enemies. Females locate and evaluate potential hosts using a combination of visual cues and long-range olfactory signals (13). At close range, they integrate chemical information detected by the antennae, legs, mouthparts, and ovipositor before making final oviposition decisions (14–17). Collectively, these chemical signals are known as semiochemicals and include both attractants and repellents; compounds that specifically deter egg laying by gravid females are often termed oviposition deterrents (13). In principle, therefore, a push–pull system can be achieved using cultivars that differ in their attractiveness or repellence to an insect pest.

Recently, we showed that potato cultivars differ markedly in their effects on the performance and oviposition preference of the potato tuber moth, *Phthorimaea operculella* (Lepidoptera: Gelechiidae) (18), a major pest of solanaceous crops worldwide (19, 20). Although larvae can develop on several *Solanum* species and other Solanaceae, field surveys and laboratory experiments indicate that potato and tobacco are the primary hosts, whereas other solanaceous plants generally support lower survival and reproduction (19, 20). Even within potato (*Solanum tuberosum*), cultivars vary substantially in susceptibility: Previous studies report wide differences in larval survival, development time, fecundity, and damage, ranging from highly susceptible to highly resistant germplasm (21–23).

Here, we evaluated the performance of the potato tuber moth across dozens of potato cultivars, identified susceptible and resistant cultivars, and searched for putative attractants and repellents underlying female oviposition preference. Using an integrated approach combining field experiments, electrophysiology, behavioral assays, and CRISPR/Cas-mediated gene editing, we identified preferred and nonpreferred cultivars and the key semiochemicals mediating attraction and repellence. We further deorphanized six odorant receptors (ORs), including three that respond specifically to the identified repellents and attractants, and demonstrated that OR knockout moths are electrophysiologically and behaviorally insensitive to the corresponding semiochemicals. Together, our findings advance understanding of the chemical ecology of the potato tuber moth and provide a mechanistic foundation for developing a push–pull strategy based on preferred and nonpreferred cultivars and/or behaviorally active semiochemicals targeting gravid females.

## Results and Discussion

### Field Survey of Adult and Larval *P. operculella* Across Potato Cultivars and Associated Damage.

In 2023, we conducted a field survey of 46 potato cultivars developed by seven Chinese breeding institutions in Daitian Shengba, Yunnan, China (Dataset S1). Each cultivar was planted in three replicate plots, for a total of 138 plots. We assessed *P. operculella* larval density (*SI Appendix*, Fig. S1*A*), tuber damage index (*SI Appendix*, Fig. S1*B*), and tuber infestation rate by larvae (*SI Appendix*, Fig. S1*C*). Based on these results, we selected eight cultivars for detailed comparison, representing cultivars with the highest and lowest larval densities (*SI Appendix*, Fig. S1*A*), damage indices (*SI Appendix*, Fig. S1*B*), and larval infestation levels (*SI Appendix*, Fig. S1*C*). Specifically, we selected Jingzhang1-4 (hereafter referred to as Jing1-4) and G8, G12, G22, and G26, all developed by Hebei Agricultural University (Dataset S1). Although cultivar 65-1 exhibited low insect density (*SI Appendix*, Fig. S1*A*), it was not included in subsequent analyses due to limited availability of tubers for follow-up experiments. Moreover, the selected panel already included cultivar G22, which showed comparably low infestation levels.

**Fig. 1.**
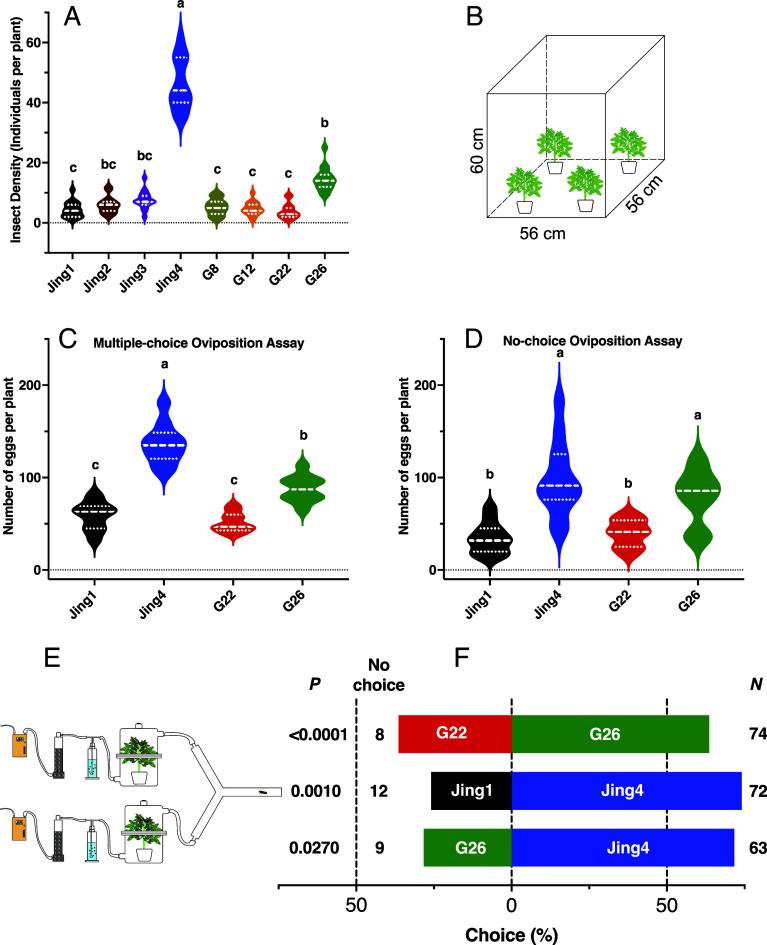
Field infestation and cultivar-specific oviposition preference of *P*. *operculella* on potato. (*A*) Larval density (insects per plant) on eight representative cultivars selected from a 46-cultivar field survey conducted under natural infestation. Violin plots show the distribution of values across five survey sites per cultivar; dashed white lines indicate medians, and dotted lines denote interquartile ranges. (*B*) Schematic of the mesh oviposition cage (56 × 56 × 60 cm) used for whole-plant oviposition assays. (*C*) Multiple-choice oviposition assay in which cultivars Jing1, Jing4, G22, and G26 were presented simultaneously (*N* = 10 replicates per cultivar). Violin plots show the number of eggs laid per plant. (D) No-choice oviposition assay in which each cage contained a single cultivar (*N* = 12 replicates per cultivar). Different letters above plots indicate significant differences among cultivars (ANOVA with Tukey’s post hoc test). (*E*) Schematic of the Y-tube olfactometer used to assess female attraction to plant odor cues. (*F*) Binary choice tests in which gravid females selected between pairs of cultivars. Bars indicate the proportion of responding females. The numbers of responding and nonresponding females are shown to the *Right* and *Left* of each bar, respectively. *P* values were calculated using a two-tailed χ^2^ test against an expected 50:50 distribution (α = 0.05).

Among the eight selected cultivars, Jing4 exhibited a significantly higher larval density than all others (Fig. 1*A*; *N* = 15, Tukey’s HSD, *P* < 0.0001) and G26 displayed a moderate insect density, whereas Jing1, G8, G12, and G22 showed the lowest insect densities. Consistent with these patterns, Jing4 sustained the most severe damage (*SI Appendix*, Fig. S2*A*), while G8 and G22 exhibited significantly lower levels of pest damage (*SI Appendix*, Fig. S2*A*). Taken together, these results, along with tuber availability for subsequent experiments, informed the selection of four cultivars for further field and laboratory assessments: two resistant cultivars (Jing1 and G22) and two susceptible cultivars (Jing4 and G26).

Field experiments conducted in Longdong, Yunnan, China, in 2024, with three replicates per cultivar, corroborated these findings. Jing4 exhibited significantly higher insect densities than all other cultivars (*SI Appendix*, Fig. S2*B*), G26 showed intermediate infestation levels, and Jing1 and G22 consistently harbored the lowest insect densities (*SI Appendix*, Fig. S2*B*) (*N* = 15, one-way ANOVA followed by Tukey’s HSD, *P* < 0.0001).

### Laboratory Assessment of Susceptible and Resistant Cultivars.

For the multiple-choice oviposition assay, the arena (Fig. 1*B*) consisted of four pots (each with one of the four disease-free cultivars), with the soil surface covered with aluminum foil. For each replicate, 2-d-old virgin adults (10 females and 12 males) were released into each cage, and oviposition was recorded after 72 h. The mean number of eggs laid per plant (Fig. 1*C*) was significantly higher on Jing4 than on all other cultivars, followed by G26, whereas Jing1 and G22 received significantly fewer eggs (N = 10, F = 63.33, *P* < 0.0001; repeated-measures one-way ANOVA with Geisser–Greenhouse correction and Tukey’s post hoc test), mirroring patterns observed in field surveys (*SI Appendix*, Fig. S2*B*).

In a no-choice assay, conducted using a similar experimental design but with cages containing only one potted plant, Jing4 and G26 harbored significantly more eggs than Jing1 and G22 (*N* = 12, F = 15.43, *P* < 0.0001; ordinary one-way ANOVA with Tukey’s post hoc test) (Fig. 1*D*).

Next, we assessed olfactory-based cultivar attractiveness using a Y-olfactometer (Fig. 1*E*) to exclude potential effects of leaf morphology and other visual cues. Individual 2 to 3-d-old females (*N* > 63), tested 24 to 48 h postmating, were assayed for 5 min. In direct comparisons between the two G cultivars, females exhibited a significant preference for G26 over G22 (*P* = 0.0270) (Fig. 1*F*; χ^2^ = 12.52, *N* = 54). Similarly, direct comparison of the two Jing cultivars showed that Jing4 attracted significantly more mated females than Jing1 (*P* = 0.0010; χ^2^ = 11.27, *N* = 60) (Fig. 1*F*). Finally, when the two most attractive cultivars were compared directly, females responded significantly more strongly to Jing4 than to G26 (χ^2^ = 4.91, *P* = 0.027; *N* = 66) (Fig. 1*F*). Collectively, these results indicate that gravid *P. operculella* females are preferentially attracted to cultivars Jing4 and G26, whereas Jing1 and G22 likely lack attractive olfactory cues and/or emit repellent volatiles.

### Prospecting for Attractants and Repellents From Potato Cultivars.

To identify putative attractant and repellent compounds, four potato cultivars (Jing1, Jing4, G22, and G26) were grown in a greenhouse, and volatile organic compounds (VOCs) emitted 30 d postplanting were analyzed by gas chromatography–mass spectrometry (GC–MS). Across all cultivars, more than 1,000 VOCs were identified and quantified using an internal standard (Dataset S2), with terpenoids and esters representing the predominant compound classes (*SI Appendix*, Fig. S3).

Unsupervised principal component analysis (PCA) of all samples showed that the first two components (PC1 and PC2) explained 57.33% and 23.26% of the total variance, respectively. The PCA score plot (*SI Appendix*, Fig. S4) revealed four well-separated and tightly clustered groups corresponding to the four cultivars, indicating pronounced between-cultivar differences and high within-group reproducibility. Notably, Jing1 and Jing4 clustered on the right side of the plot, whereas G22 and G26 clustered on the left.

Guided by this clustering, we next performed supervised pairwise differential-abundance analyses within each cultivar pair to identify VOCs with a fold change greater than 2 and a false discovery rate (FDR) below 0.05. These analyses identified 392 compounds enriched in G22 relative to G26 and 20 compounds enriched in G26 relative to G22 (Dataset S3). Similarly, 233 VOCs were enriched in Jing1 compared with Jing4, whereas 14 VOCs were enriched in Jing4 compared with Jing1.

**Fig. 2.**
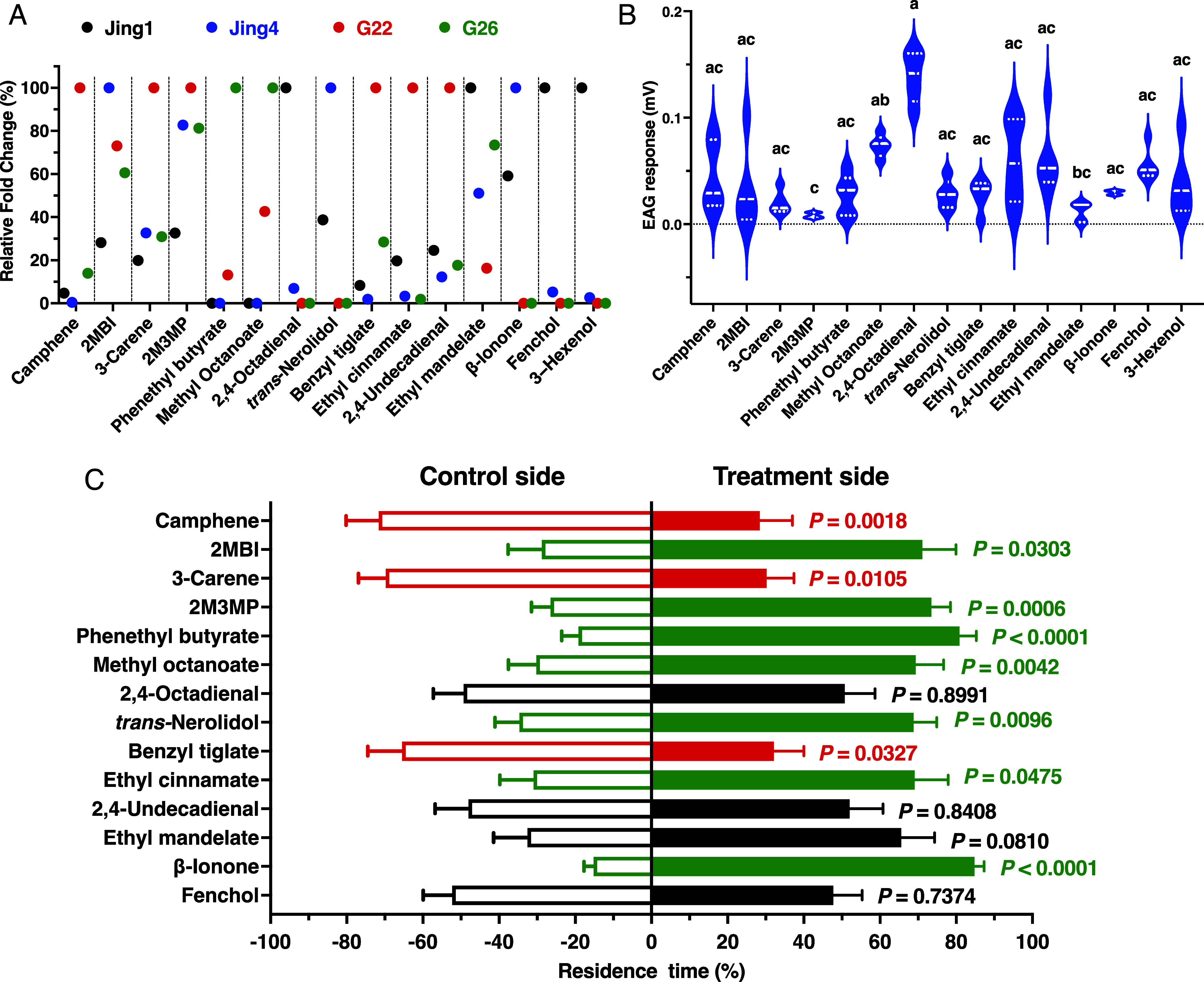
Cultivar-specific volatile profiles and olfactory responses of *P. operculella* to candidate VOCs. (*A*) Relative fold change of 15 odorants in susceptible and resistant cultivars. For brevity, 2-methylbutyl isovalerate and 2-methyl-3-methylpyrazine are abbreviated in this figure as 2MBI and 2M3MP, respectively. (*B*) EAG responses (mean ± SE) of female antennae to the 15 VOCs. Different letters above plots indicate significant differences among cultivars (Friedman test with Dunn’s multiple-comparisons post hoc test; *F* = 43.54). (*C*) Behavioral responses of gravid females to 14 cultivar-associated VOCs in a four-arm olfactometer. Bars show the residence time (mean ± SEM, N = 20 to 25) in the odor-treated arm (solid bars) versus a paraffin-oil control (empty bars).

We next selected 12 VOCs for electrophysiological and behavioral assays based on their differential abundance between cultivars (Fig. 2*A* and Dataset S3). These compounds were camphene: Jing1/Jing4, 11.2-fold; G22/G26, 7.0-fold; ethyl cinnamate: Jing1/Jing4, 5.9-fold; G22/G26, 51.7-fold; benzyl tiglate: Jing1/Jing4, 4.3-fold; G22/G26, 3.4-fold; nerolidol (=1,6,10-dodecatrien-3-ol, 3,7,11-trimethyl-): Jing4/Jing1, 6.9-fold; 3-carene: G22/G26, 3.2-fold; 2-methoxy-3-methylpyrazine (=pyrazine, 2-methoxy-3-methyl-): Jing1/Jing4, 3.9-fold; phenethyl butyrate (=butanoic acid, 1-phenylethyl ester): Jing1/Jing4, 2.4-fold, G22/G26, 3.2-fold; methyl octanoate (=octanoic acid, methyl ester): G26/G22, 2.5-fold; 2,4-octadienal (=2,4-octadienal, (*E,E*)-): Jing1/Jing4, 14.6-fold; 2,4-undecadienal (=(*E,*E)-2,4-undecadienal): G22/G26, 5.6-fold; and fenchol: Jing1/Jing4, 19.9-fold. Ethyl mandelate was also selected based on its enrichment in G26 relative to G22 (9.1-fold). In addition, we included three VOCs of biological interest (Dataset S3) that did not meet the above statistical criteria. These were β-ionone, which showed a 1.7-fold enrichment in Jing4 relative to Jing1 and has been reported as an attractant for male *Glyphodes pyloalis* moths (24); 2-methylbutyl isovalerate, which has been shown to be electroantennographically active in nonmoth insect species (25); and (*Z*)-3-hexenol, which was highly enriched in Jing1 relative to Jing4 (37-fold) but narrowly failed the FDR cutoff (FDR = 0.06) and has been reported as an EAG-active oviposition deterrent for *P. operculella* (26). Our selected panel of odorants thus includes 4 VOCs enriched in Jing1, 3 in Jing4, 6 in G22, and 2 in G26 (Fig. 2*A*).

### Electrophysiological and Behavioral Measurements of Putative Attractants and Repellents.

To streamline the compound panel, we recorded electroantennogram (EAG) responses from female antennae. All tested compounds elicited significant EAG responses (Fig. 2*B*). Among them, 2,4-octadienal induced significantly stronger responses than all other compounds, including the positive control 3-hexenol (Friedman test with Dunn’s multiple-comparisons post hoc test; *F* = 43.54). Despite these differences, EAG profiling did not allow further reduction of the compound panel for subsequent behavioral assays using a multichoice olfactometer (27). The only exception was 3-hexenol, which was excluded because it is already known to act as an oviposition deterrent for *P. operculella* (26). We also compared male and female EAG responses to this panel of semiochemicals (*SI Appendix*, Fig. S5). Male antennae showed significantly higher responses (Mann–Whitney *U* test, *N* = 5) to 2-methylbutyl isovalerate, 2-methoxy-3-methylpyrazine, methyl octanoate, and ethyl mandelate. By contrast, camphene, ethyl cinnamate, and β-ionone elicited stronger responses from female than male antennae.

Despite eliciting the strongest EAG responses, 2,4-octadienal did not induce a significant behavioral preference, as females showed no difference in residence time between the treatment and control sides of the arena (*SI Appendix*, Fig. S6) (*P* = 0.8991; Mann–Whitney *U* test.) Similarly, residence times in the presence of 2,4-undecadienal (*P* = 0.8408), ethyl mandelate (*P* = 0.0810), and fenchol (*P* = 0.7374) did not differ significantly from their respective controls (black bars in Fig. 2*C*). We therefore conclude that these four VOCs do not function as attractants or repellents for mated *P. operculella* females under the conditions tested. In contrast, camphene, 3-carene, and benzyl tiglate elicited behavioral responses consistent with repellency. Female residence times were significantly shorter in the presence of camphene (*P* = 0.0018), 3-carene (*P* = 0.0105), and benzyl tiglate (*P* = 0.0327) than in the corresponding controls (red bars in Fig. 2*C*). Notably, all three compounds are enriched in cultivar G22 (Fig. 2*A*), which also exhibited oviposition deterrence in both multiple-choice and no-choice assays (Fig. 1). These results suggest that these VOCs may contribute, at least in part, to the oviposition deterrence observed for this cultivar. Conversely, 2-methylbutyl isovalerate, 2-methoxy-3-methylpyrazine, phenethyl butyrate, methyl octanoate, *trans*-nerolidol, ethyl cinnamate, and β-ionone elicited significant attraction responses, as indicated by longer residence times in treatment arms compared with controls (*P* = 0.0303, 0.0006, <0.0001, 0.0042, 0.0096, 0.0475, and <0.0001, respectively; green bars in Fig. 2*C*). Among these, 2-methylbutyl isovalerate, *trans*-nerolidol, and β-ionone are enriched in cultivar Jing4 (Fig. 2*A*), suggesting that these attractant VOCs may underlie the enhanced attraction and oviposition preference observed for this cultivar relative to others (Fig. 1). Collectively, these findings motivated us to survey the *P. operculella* genome to identify candidate ORs potentially involved in the detection of these attractants and repellents.

### Prospecting for ORs Sensitive to Attractants and Repellents.

**Fig. 3.**
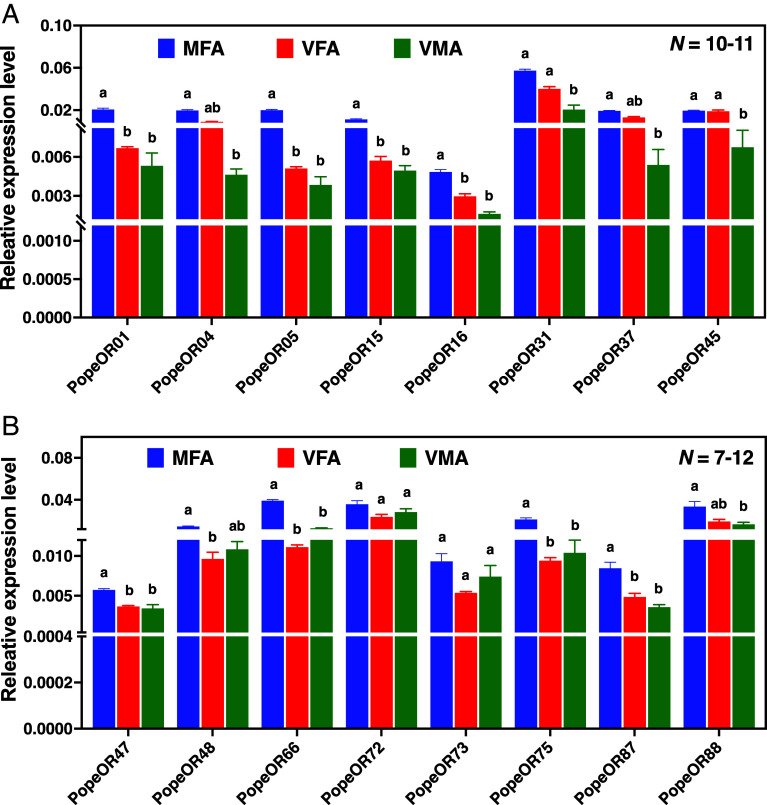
Expression profiles of candidate *PopeOR* genes in *P. operculella*. (*A*) Relative transcript levels of *PopeOR01, PopeOR04, PopeOR05, PopeOR15, PopeOR16, PopeOR31, PopeOR37,* and *PopeOR45*. (*B*) Relative transcript levels of *PopeOR47, PopeOR48, PopeOR66, PopeOR72, PopeOR73, PopeOR75, PopeOR87,* and *PopeOR88*. For each gene, expression was quantified by qRT–PCR in mated female antennae (MFA), virgin female antennae (VFA), and virgin male antennae (VMA. Bars show mean ± SEM from three biological replicates. Different letters above bars indicate significant differences among tissues within each gene (one-way ANOVA followed by Tukey’s HSD test, *P* < 0.05).

Previously, we identified the OR coreceptor Orco and 92 putative OR genes in the *P. operculella* genome (28). Based on transcriptome analyses (28), we selected 30 female-biased *OR* genes, together with *PopeOrco*, for quantitative expression profiling (Dataset S4). Consistent with their roles in olfactory perception, the majority of *PopeOR* genes, except PopeOR71, were expressed at higher levels in antennae than in other tissues examined (*SI Appendix*, Figs. S7 and S8). Except for *PopeOR15* and *PopeOR31* (*SI Appendix*, Fig. S7), all receptors were expressed in both adult and larval tissues (*SI Appendix*, Fig. S7 and S8). Like *PopeOrco* (VMA/VFA, *P* = 0.0019; VMA/MFA, *P* = 0.0486, Student’s *t* test), *PopeOR64* (*SI Appendix*, Fig. S8) exhibited significantly higher expression in male antennae than in female antennae (VMA/VFA and VMA/MFA, *P* < 0.001, Student’s *t* test), suggesting a potential role in sex-pheromone detection. In addition, *PopeOR42*, *PopeOR46, PopeOR63*, and *PopeOR64* were downregulated in antennae of mated females (*SI Appendix*, Fig. S8). From this dataset, we selected 16 OR genes that appeared to be upregulated in mated compared with virgin female antennae, i.e., *PopeOR01, 4, 5, 15, 16, 31, 37, 45, 47, 48, 66, 72, 73, 75, 87*, and *88*, for further analysis. Among these, *PopeOR01, 5, 15, 16, 47, 66, 75*, and *87* showed significantly higher transcript levels in mated than in virgin female antennae (Fig. 3), suggesting that these receptors may be involved in the detection of oviposition-related attractants or deterrents. Based on the other *OR* genes that were not downregulated, we then sought to deorphanize all these 16 ORs using a panel of 121 odorants, including the 15 VOCs selected based on their differential abundance among potato cultivars and previously reported Solanaceae volatiles, to identify ligands potentially underlying the observed behavioral responses.

### Functional Characterization of OR for Behaviorally Active VOCs.

To deorphanize these 16 receptors, we first cloned and subcloned the corresponding genes into pGEMHE, using primers designed to include a Kozak consensus sequence (Dataset S5): see *SI Appendix*, *SI Text* for GenBank accession numbers. *PopeOR01*, *PopeOR04, PopeOR05, PopeOR15, PopeOR16, PopeOR31, PopeOR37, PopeOR45, PopeOR47, PopeOR48, PopeOR66, PopeOR72, PopeOR73, PopeOR75, PopeOR87,* and *PopeOR88.* Next, each OR was coexpressed with the coreceptor Orco *PopeOrco* in the *Xenopus* oocyte recording system and challenged with a panel of 121 odorants (Dataset S6), including electrophysiologically and behaviorally active compounds.

**Fig. 4.**
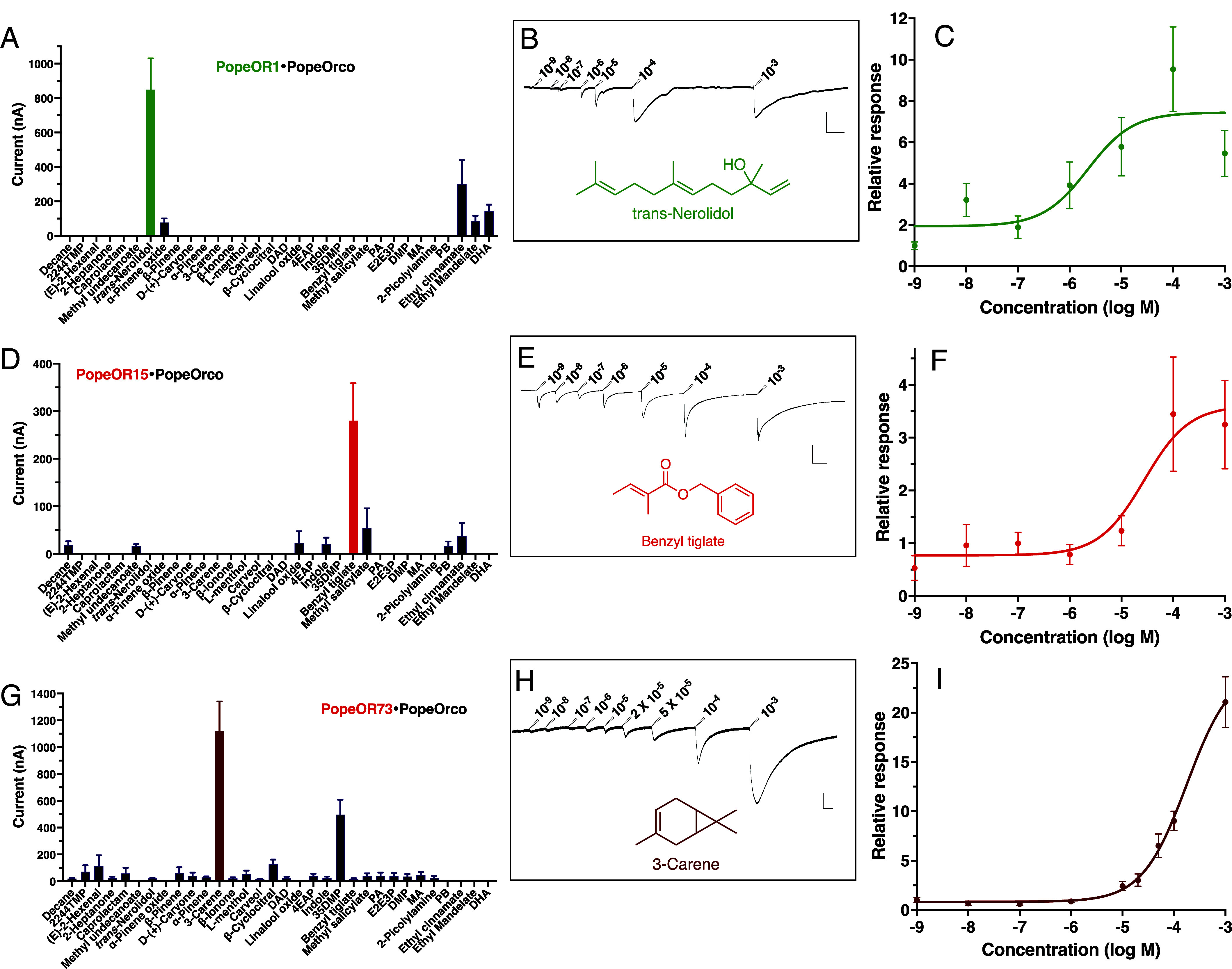
Functional characterization of PopeOR01, PopeOR15, and PopeOR73 by two-electrode voltage clamp in *Xenopus laevis* oocytes. (*A*) Odorant response spectrum of PopeOR01 coexpressed with PopeOrco in *X. laevis* oocytes. Bars show mean current amplitudes (nA) elicited by individual volatile compounds. Data are presented in mean ± SEM. (*B*) Representative two-electrode voltage-clamp trace from an oocyte expressing PopeOR01 and PopeOrco in response to increasing concentrations of nerolidol (10^−9^ to 10^−^^3^ M, as indicated above each pulse). (Scale for horizontal bar, 2 min; vertical bar, 500 nA.) (*C*) Normalized dose–response curve for PopeOR01 challenged with nerolidol. Points represent mean relative responses ± SEM, fitted with a sigmoidal function to estimate an EC_50_ of 2.25 × 10^−6^ M. (*D*) Odorant response spectrum of PopeOR15 coexpressed with PopeOrco in *Xenopus* oocytes. (*E*) Representative voltage-clamp trace from an oocyte expressing PopeOR15 and PopeOrco, stimulated with increasing concentrations of benzyl tiglate (10^−9^ to 10^−3^ M). (Scale for horizontal bar, 2 min; vertical bar, 500 nA.) (*F*) Normalized dose–response curve for PopeOR15 in response to benzyl tiglate. Mean relative responses ± SEM are shown; curve fitting yields an EC_50_ of 2.74 × 10^−^^5^ M. (*G*) Odorant response spectrum of PopeOR73/PopeOrco-expressing oocytes. (*H*) Representative voltage-clamp trace from an oocyte expressing PopeOR73 and PopeOrco, stimulated with increasing concentrations of 3-carene (10^−9^ to 10^−^^3^ M; intermediate doses indicated). (Scale for horizontal bar, 1 min; vertical bar, 200 nA.) (*I*) Normalized dose–response curve for PopeOR73 challenged with 3-carene. Points represent mean relative responses ± SEM, fitted with a sigmoidal function to estimate an EC_50_ of 1.7 × 10^−^^4^ M. The following abbreviations were used in this figure: 2244TMP, 2,2,4,4-tetramethylpentane; MU, methyl undecanoate; DAD, dihydroactinidiolide; 4EAP, 4-ethylacetophenone; 35DMP, 3,5-dimethylphenol; PA, phenylacetaldehyde; E2E3P, ethyl (2*E*)-3-phenylacrylate (=ethyl cinnamate); DMP, dimethyl phthalate; MA, methyl anthranilate; PB, phenethyl butyrate; DHA, dihydroxyacetophenone.

Oocytes coexpressing PopeOrco with *PopeOR04, 16, 31, 37, 45, 48, 72, 75, 87,* or *88* showed no responses to any tested compounds. In contrast, PopeOR5/PopeOrco-expressing oocytes elicited the strongest currents in response to methyl 2-methoxybenzoate, with moderate responses to *trans*-nerolidol, ethyl cinnamate, and hexanal (*SI Appendix*, Fig. S9*A*). Similarly, PopeOR47 responded to α-terpinene, α-pinene oxide, camphene, methyl octanoate, 4-hydroxybenzyl alcohol, 4-hydroxyphenylacetic acid, and undecylenic acid (*SI Appendix*, Fig. S9*B*), whereas PopeOR66 (*SI Appendix*, Fig. S9*C*) was activated by 3-methyl-1-phenyl-3-pentanol, 3,4-dimethoxytoluene, undecylenic acid, dodecane, 3-hexanol, and linalool. However, the response profiles of these receptors did not reveal clear ligand specificity. By contrast, three ORs exhibited robust and selective responses to behaviorally active VOCs from potato cultivars. PopeOR01/PopeOrco-expressing oocytes showed strong, dose-dependent responses to the attractant *trans*-nerolidol (Fig. 4 *A*–*C* and *SI Appendix*, Fig. S10*A*), with only weak responses to α-pinene oxide, ethyl cinnamate, ethyl mandelate, and dihydroxyacetophenone (Fig. 4*A*). PopeOR1*5* responded in a dose-dependent manner to the repellent benzyl tiglate (Fig. 4 *D*–*F* and *SI Appendix*, Fig. S10*B*), while other compounds, including ethyl cinnamate, elicited only minor currents. Finally, PopeOR73 was selectively activated by 3-carene in a dose-dependent fashion (Fig. 4 *G*–*I* and *SI Appendix*, Fig. S10*C*) and showed a moderate response to 3,5-dimethylphenol (Fig. 4*G*). To unambiguously determine the roles of these receptors in detecting behaviorally active VOCs emitted by potato cultivars, we next sought to knock out the corresponding OR genes and compare the behavioral responses of knockout and wild-type (WT) female moths.

### Knockout Moths Are Insensitive to the Attractant *trans*-Nerolidol and the Repellent 3-Carene.

**Fig. 5.**
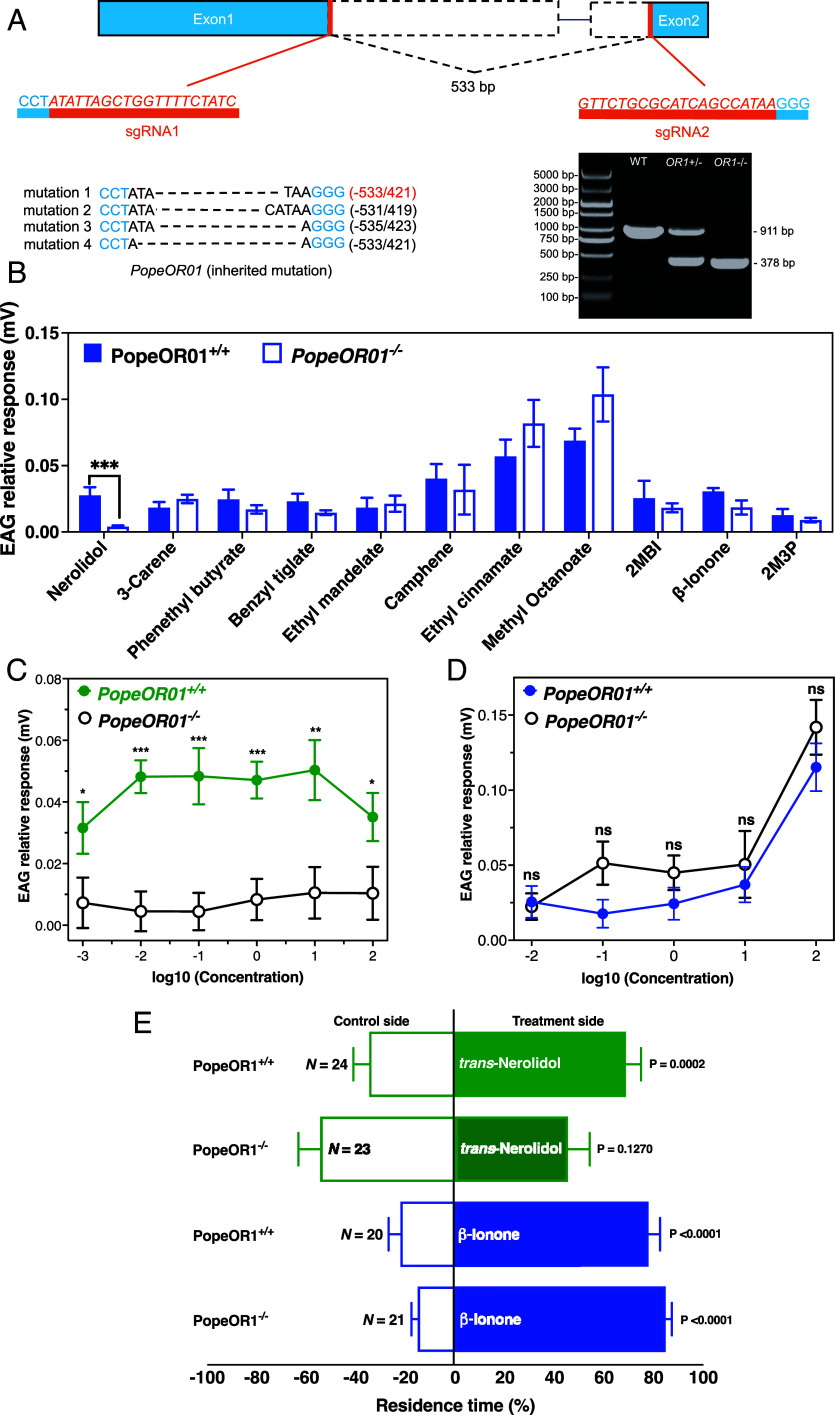
CRISPR/Cas9 knockout of PopeOR01 specifically impairs nerolidol detection and attraction. (*A*) Schematic of CRISPR/Cas9 editing of PopeOR01. Two sgRNAs targeting exon 1 and exon 2 generated a 533-bp deletion. Representative genotyping PCR (*Right*) shows the WT band (911 bp), the heterozygous pattern, and the shorter mutant band (378 bp) in homozygous PopeOR01^−/−^ individuals. The four inherited indel alleles are listed below the schematic. (*B*) EAG responses of WT and *PopeOR01^−/−^* females to 11 behaviorally active volatile compounds. Bars show relative response amplitudes (mV; mean ± SEM). Among all tested VOCs, only nerolidol evoked significantly weaker responses in PopeOR01^−/−^ antennae than in WT, whereas responses to the other compounds were comparable between genotypes. (*C*) Dose–response curves for nerolidol in WT and PopeOR01^−/−^ females. EAG responses (mean ± SEM) are plotted against log_10_ concentration (0.001 to 100 mg/mL; log_10_ = −3 to 2). At most doses, PopeOR01^−/−^ antennae showed significantly reduced responses compared with WT (*P* values indicated above points; *P < 0.05, ***P < 0.001; Student’s *t* test at 0.01 mg/mL (*P* < 0.0001); Mann–Whitney *U* test at 0.001 mg/mL (*P* = 0.0428), 0.1 mg/mL (*P* < 0.0001), 1 mg/mL (*P* < 0.0001), 10 mg/mL (*P* = 0.0012), and 100 mg/mL (*P* = 0.0226). (*D*) Dose–response curves for β-ionone in WT and PopeOR01^−/−^ females. EAG amplitudes increased with concentration in both genotypes, but no significant differences were detected at any dose (Mann–Whitney *U* test at 0.001—10 mg/mL; Student’s *t* test at 100 mg/mL, all not significant.) (*E*) Four-arm olfactometer assays testing behavioral responses of WT and PopeOR01^−/−^ females to nerolidol and β-ionone. Bars show the proportion of residence time (mean ± SEM, N = 20 to 24) in the odor arm (solid bars) versus the paraffin-oil control arm (empty bars). Mann–Whitney *U* test was used for nerolidol (PopeOR01^−/−^, *P* > 0.05; PopeOR01^+/+^, *P* = 0.0002), and Student’s *t* test was used for β-ionone (both genotypes, *P* < 0.0001). WT females were strongly attracted to both nerolidol and β-ionone, whereas PopeOR01^−/−^ females lost attraction to nerolidol but retained attraction to β-ionone.

CRISPR/Cas9-mediated mutagenesis (Dataset S7) successfully generated a homozygous *PopeOR01*^−/−^ line. Sequencing revealed a 533-bp deletion spanning exons 1 and 2, and PCR analysis confirmed the loss of the WT allele (911 bp) and the presence of a truncated mutant fragment (378 bp) in knockout individuals (Fig. 5*A*). To assess the role of *PopeOR01* in antennal olfactory detection, we recorded EAG responses to a panel of 10 volatile compounds that exhibited significant attractive or repellent effects in behavioral assays, together with ethyl mandelate (Fig. 2*C*). Among these behaviorally active compounds, only *trans*-nerolidol elicited markedly reduced EAG responses in *PopeOR01*^−/−^ antennae compared with WT antennae; responses to all other tested compounds were comparable to, or slightly higher than, those of WT moths (Student’s *t* test, *N* = 5 to 10) (Fig. 5*B*).

Concentration–response analyses further showed that WT females exhibited robust, dose-dependent EAG responses to *trans*-nerolidol, whereas *PopeOR01*^−/−^ females responded weakly across all tested concentrations (*N* = 21 to 28; Student’s *t* test and Mann–Whitney *U* test) (Fig. 5*C*). In contrast, dose–response curves for β-ionone did not differ between genotypes (Fig. 5*D*), indicating that *PopeOR01* is specifically required for nerolidol, but not β-ionone, detection at the antennal level.

Behavioral assays were consistent with these electrophysiological findings. WT females spent significantly more time in the arena containing *trans*-nerolidol than in the solvent control, whereas *PopeOR01*^−/−^ females showed no significant preference for *trans*-nerolidol over paraffin oil (Fig. 5*E*; Mann–Whitney *U* test, *P* = 0,1270). By contrast, both WT and knockout females were strongly attracted to β-ionone (Fig. 5*E*; Student’s *t* test, *P* < 0.0001). Together, these results demonstrate that *PopeOR01* is selectively tuned to nerolidol and is essential for nerolidol-mediated attraction, while being dispensable for β-ionone detection and associated behavioral responses.

**Fig. 6.**
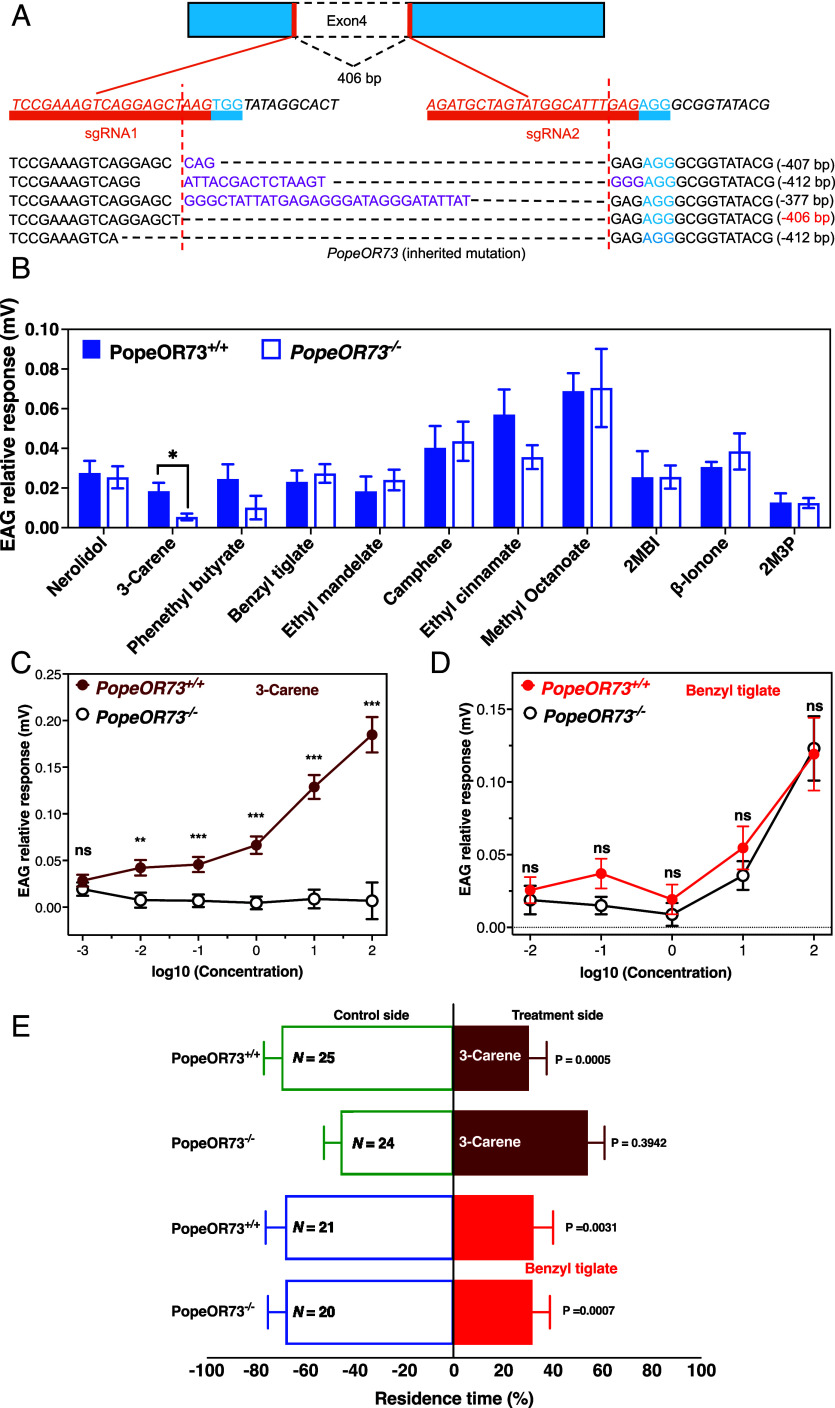
CRISPR/Cas9 knockout of PopeOR73 specifically impairs 3-carene detection and avoidance. (*A*) Schematic of CRISPR/Cas9 editing of PopeOR73. Two sgRNAs targeting exon 4 generated a 406-bp deletion. The inherited mutant alleles are listed below the schematic. (*B*) EAG responses of WT and PopeOR73^−/−^ females to 11 behaviorally active volatile compounds. Bars show relative response amplitudes (mV; mean ± SEM). Among all tested VOCs, only 3-carene evoked significantly weaker responses in PopeOR73^−/−^ antennae than in WT (*P* < 0.05), whereas responses to the other compounds were comparable between genotypes (Student’s *t* test was used to compare EAG amplitudes between PopeOR73^+/+^ and PopeOR73^−/−^ females in response to 3-carene, *P* = 0.0287). (*C*) Dose–response curves for 3-carene in WT and PopeOR73^−/−^ females. EAG responses (mean ± SEM) are plotted against log_10_ ed (0.001 to 100 mg/mL; log_10_C = –3 to 2). At most doses, PopeOR73^−/−^ antennae showed significantly reduced responses compared with WT (*P* values indicated above points; *P < 0.01, **P < 0.001; ns, not significant; Mann–Whitney *U* test was used at 0.01 mg/mL (*P* = 0.0057) and at 0.1 to 1 and 100 mg/mL (*P* < 0.0001), whereas Student’s *t* test was used at 10 mg/mL, *P* < 0.0001). (*D*) Dose–response curves for benzyl tiglate in WT and PopeOR73^−/−^ females. EAG amplitudes increased with concentration in both genotypes, but no significant differences were detected at any dose (ns; Mann–Whitney *U* test at 0.01 to 1 mg/mL; Student’s *t* test at 1 to 10 mg/mL; all not significant). (*E*) Four-arm olfactometer assays testing behavioral responses of WT and PopeOR73^−/−^ females to 3-carene and benzyl tiglate. Bars show the proportion of residence time (mean ± SEM, N = 20 to 25) in the odor arm (solid bars) versus the paraffin-oil control arm (empty bars). Student’s *t* test at PopeOR73^−/−^ females toward 3-carene (*P* = 0.3942) and benzyl tiglate (*P* = 0.0007); Mann–Whitney *U* test at PopeOR73+/+ females toward 3-carene (*P* = 0.0005) and benzyl tiglate (*P* = 0.0031). WT females avoided both 3-carene and benzyl tiglate, whereas PopeOR73^−/−^ females lost avoidance to 3-carene but retained avoidance to benzyl tiglate.

A homozygous PopeOR73^−/−^ line was generated by CRISPR/Cas9-mediated genome editing, resulting in a 406-bp deletion within exon 4 (Fig. 6*A*). To investigate the role of PopeOR73 in olfactory detection, we recorded EAG responses to a panel of 11 volatile compounds, including 10 odorants that elicited significant attractive or repellent effects in behavioral assays, as well as ethyl mandelate (Fig. 2*C*). Among these compounds, only 3-carene elicited significantly reduced antennal responses in PopeOR73^−/−^ females compared with WT females (Student’s *t* test, *P* = 0.0287), whereas responses to the remaining compounds did not differ between genotypes (Fig. 6*B*).

Concentration–response analyses further demonstrated that PopeOR73^−/−^ moths exhibited markedly attenuated antennal sensitivity to 3-carene relative to WT moths (Fig. 6 *C* and *D*). At the lowest concentration tested (0.001 mg/mL), EAG responses did not differ significantly between genotypes (Student’s *t* test, *P* > 0.05). However, from 0.01 mg/mL onward, WT moths showed significantly higher EAG amplitudes than the knockout line (Mann–Whitney *U* test, *P* = 0.0057 at 0.01 mg/mL; *P* < 0.0001 at 0.1 to 1 and 100 mg/mL; Student’s *t* test, *P* < 0.0001 at 10 mg/mL). In contrast, dose–response curves for benzyl tiglate were indistinguishable between PopeOR73^+/+^ and PopeOR73^−/−^ antennae at all tested concentrations (Fig. 6*D*; Mann–Whitney *U* test at 0.01 to 1 mg/mL; Student’s *t* test at 1 to 10 mg/mL; all not significant), indicating that PopeOR73 is not required for benzyl tiglate detection at the antennal level.

Behavioral assays corroborated these physiological findings. WT females exhibited significant avoidance responses to both 3-carene and benzyl tiglate, confirming their repellent properties. In contrast, PopeOR73^−/−^ females completely lost avoidance behavior toward 3-carene (Student’s *t* test, *P* = 0.3942), while maintaining normal avoidance of benzyl tiglate (Student’s *t* test, *P* = 0.0007) (Fig. 6*E*). Together, these results demonstrate that PopeOR73 is specifically required for the antennal detection and behavioral avoidance of 3-carene but does not participate in the perception of benzyl tiglate.

### Oviposition Preferences in OR Mutants.

**Fig. 7.**
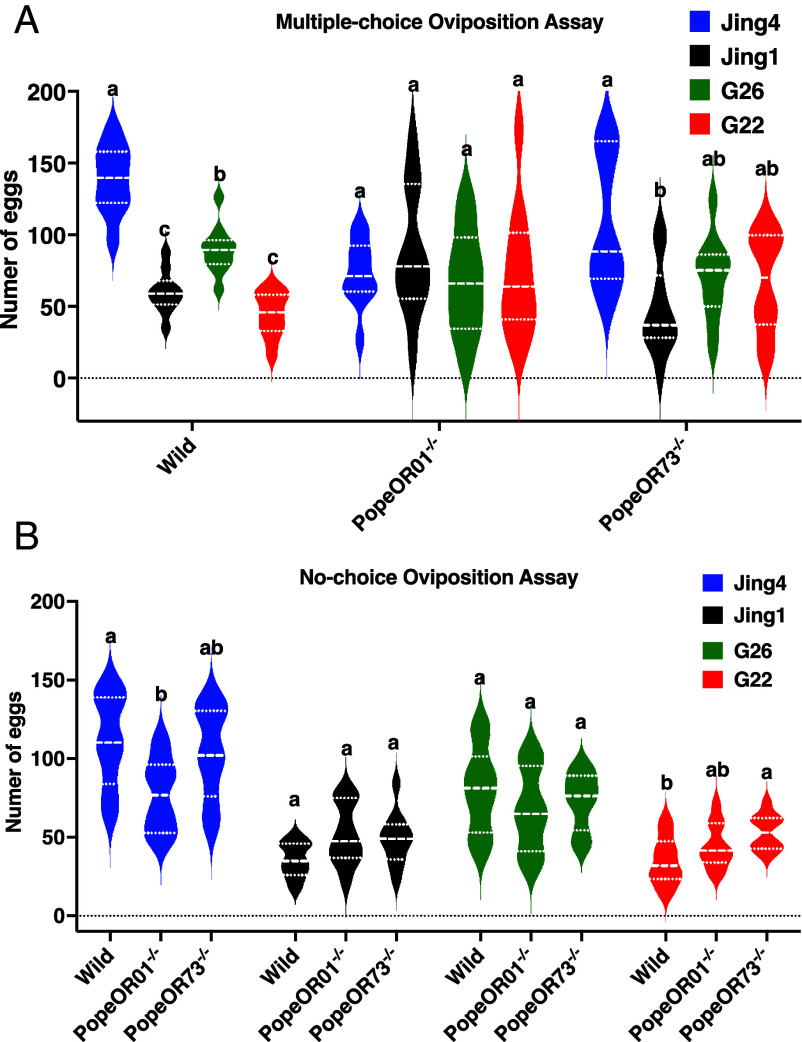
Oviposition preference of WT and OR mutants among potato cultivars. (*A*) Multiple-choice oviposition assay in which cultivars Jing1, Jing4, G22, and G26 were presented simultaneously (*N* = 10 replicates per cultivar). Violin plots show the number of eggs laid per plant by WT, *PopeOR01*^−/−^, and *PopeOR73*^−/−^ gravid females. (*B*) No-choice oviposition assay in which each cage contained a single cultivar (*N* = 10 replicates per cultivar). Violin plots show the number of eggs laid per plant. Different letters above plots indicate significant differences among cultivars within each genotype (*A*) or among genotypes within each cultivar (*B*) (one-way ANOVA with Tukey’s post hoc test, *P* < 0.05). Colors denote cultivars: Jing4 (blue), Jing1 (black), G26 (green), and G22 (red).

We performed additional oviposition experiments to further examine the causal link between cultivar VOC cues, specific ORs, and oviposition behavior by disentangling oviposition choice (preference/allocation) from overall fecundity. In the multichoice assay, WT females showed a clear cultivar-dependent allocation of eggs, with the highest oviposition on Jing4, intermediate levels on G26, and lower oviposition on Jing1 and G22, indicating a robust cultivar preference (*N* = 10, F = 58.818, *P* < 0.001; repeated-measures one-way ANOVA with Tukey’s post hoc test) (Fig. 7*A*). In contrast, PopeOR01^−/−^ females no longer exhibited significant differences in egg allocation among the four cultivars, demonstrating that PopeOR1 is required for cultivar-specific oviposition preference and that its loss leads to a more uniform distribution of eggs (*N* = 10, F = 0.50, *P* = 0.685; repeated-measures one-way ANOVA with Tukey’s post hoc test) (Fig. 7*A*). PopeOR73^−/−^ females retained a strong preference for Jing4, but differences among the remaining cultivars were markedly compressed, suggesting that PopeOR73 primarily contributes to fine-scale discrimination and/or the strength of avoidance among nonpreferred cultivars rather than determining the primary preference itself (*N* = 10, F = 5.114 *P* = 0.005; repeated-measures one-way ANOVA with Tukey’s post hoc test) (Fig. 7*A*).

In no-choice assays (single cultivar offered), overall egg output was broadly comparable across genotypes, arguing against a generalized reduction in fecundity due to PopeOR1 or PopeOR73 disruption. Notably, PopeOR01^−/−^ effects were cultivar dependent: Mutants tended to lay fewer eggs on Jing4 (*N* = 10, F = 4.305, *P* = 0.024; repeated-measures one-way ANOVA with Tukey’s post hoc test) (Fig. 7*B*), whereas PopeOR73^−/−^ females tended to lay more eggs on G22, consistent with the hypothesis that PopeOR73 mediates deterrent cues such as 3-carene, which is enriched in G22, and that disruption of this pathway partially relieves oviposition suppression on that cultivar (*N* = 10, F = 4.449, *P* = 0.021; repeated-measures one-way ANOVA with Tukey’s post hoc test) (Fig. 7*B*). Collectively, our results support a model in which PopeOR01 primarily drives the establishment of cultivar preference via attractive cues (e.g., nerolidol), while PopeOR73 acts mainly as a deterrent/filtration pathway via repellent cues (e.g., 3-carene); together, these pathways shape cultivar-specific oviposition allocation in WT females.

Although our results link cultivar VOCs and antennal OR function to oviposition allocation, oviposition decisions are likely shaped by multimodal inputs, including contact chemosensation via receptors in the tarsi and/or ovipositor. Thus, additional sensory appendages and chemoreceptor families may also contribute to host choice at close range (29). Moreover, while EAG provides population-level antennal sensitivity, future single-sensillum recordings and OR–ORN localization will be needed to improve spatial resolution. Finally, extending OR mutant assays to additional Solanaceae hosts and their volatile blends will help define the broader ecological relevance of the identified OR–ligand pairs.

## Conclusions

Our field and laboratory studies demonstrate that cultivar-dependent variation in *P. operculella* infestation is driven, at least in part, by differences in volatile emission profiles that differentially attract or repel gravid females. We identify specific plant-emitted VOCs, including *trans*-nerolidol, β-ionone, 3-carene, and benzyl tiglate, that function as behaviorally active attractants or repellents. Together, these findings provide a mechanistic basis for environmentally friendly potato pest management, informing the selection of insect-resistant cultivars and enabling the potential use of these semiochemicals for monitoring, attraction, or repellency of the potato tuber moth. More broadly, our results support the development of cultivar-based push–pull strategies for sustainable pest control.

To establish a broader framework for semiochemical-based management of *P. operculella*, we further identified ORs in gravid females that underlie potato cultivar susceptibility and resistance. Screening a large odorant panel enabled deorphanization of six ORs, three of which showed narrow sensitivity to key semiochemicals associated with susceptible or resistant cultivars. Notably, PopeOR01, whose expression is upregulated following mating, responded selectively to the attractant *trans*-nerolidol, a compound enriched in the highly susceptible cultivar Jing4, which exhibited the highest larval densities among the 46 cultivars surveyed. In contrast, PopeOR15 and PopeOR73 were narrowly tuned to the oviposition deterrents benzyl tiglate and 3-carene, respectively, both of which were enriched in the resistant cultivar G22, which showed consistently low insect densities and minimal egg laying. Although additional visual and olfactory cues may contribute, our results strongly suggest that *trans*-nerolidol, 3-carene, and benzyl tiglate play key roles in shaping cultivar-specific susceptibility and resistance to the potato tuber moth.

In insects, ORs function as ligand-gated ion channels that form heteromeric complexes with the conserved coreceptor Orco (30, 31). Structural and functional studies suggest that the ion-conducting pore is assembled from C-terminal subunits, comprising one ligand-binding OR and three Orco subunits (32, 33). Consistent with this model, AlphaFold3 predictions indicate that PopeOR01, PopeOR15, and PopeOR73 assemble into OR–Orco complexes (*SI Appendix*, Fig. S11 and Dataset S8) that are structurally homologous to previously characterized insect ORs (*SI Appendix*, *Supporting Text*). In particular, PopeOrco closely resembles ApisOrco (34), whereas the ligand-binding ORs show moderate structural divergence from ApisOR5 (35).

Molecular docking and molecular dynamics simulations (*SI Appendix*, Figs. S12–S14 and Dataset S8) identified 3-carene, *trans*-nerolidol, and benzyl tiglate as the highest-affinity ligands for PopeOR73, PopeOR01, and PopeOR15, respectively. In all cases, ligand binding was stable and dominated by hydrophobic and aromatic interactions, supplemented by key hydrogen bonds, in agreement with electrophysiological recordings and behavioral assays.

These structural models provide a framework for the rational discovery of ligands that activate or inhibit *P. operculella* ORs, thereby disrupting insect–plant chemical communication. Supporting this potential, CRISPR/Cas9-mediated gene knockout, combined with electrophysiological and behavioral analyses, demonstrated that PopeOR01 and PopeOR73 are essential for detecting the attractant *trans*-nerolidol and the repellent 3-carene, respectively, establishing these receptors as promising molecular targets for semiochemical-based pest management. Importantly, our additional oviposition assays further disentangle oviposition choice from fecundity and show that PopeOR01 is required for cultivar-specific oviposition preference, whereas PopeOR73 primarily modulates avoidance and fine-scale discrimination among nonpreferred cultivars; thus, distinct OR pathways integrate attractive and deterrent VOC cues to shape egg allocation across potato cultivars.

Together, our integrative chemical, genetic, structural, and behavioral analyses link potato cultivar chemistry to moth olfactory receptor function and pest outcomes, providing a molecular roadmap for the development of targeted, sustainable, and ecologically sound strategies to manage the potato tuber moth.

## Materials and Methods

### Insect Rearing.

A potato tuber moth colony was maintained at the Institute of Plant Protection, Chinese Academy of Agricultural Sciences (Beijing, China). Adults were reared in plastic jars with gauze for oviposition, and larvae were raised on potatoes in nylon cages. All insects were kept in SANYO/Panasonic incubators (model MLR-351H; Panasonic Healthcare, Japan) at 26 °C, 60% RH, and a 12:12 h light:dark photoperiod.

### Study Sites and Potato Cultivars.

In spring 2023, 46 potato cultivars from eleven institutions (Dataset S1) were tested in Datian Shengba, Yunnan, China (25.25° N, 103.63° E). Plots were arranged in a randomized block design with three replicates per cultivar, totaling 138 plots (each 13.2 m^2^; 4 × 3.3 m, 0.7 m spacing, and border rows). Surveys were conducted at ~10-d intervals (approximately 60, 70, 80, and 90 d after planting) and included counting potato tuber moth larvae on leaves (five plants randomly selected per plot); because results were similar across surveys, larval counts from the first survey (≈60 DAP) are reported. Tuber damage was assessed at harvest by sampling 50 tubers per plot. Following established damage-rating schemes for internal-boring lepidopteran pests (e.g., pink bollworm *Pectinophora gossypiella*) (36) tuber damage was scored on a 0 to 5 scale based on the number and maximum depth of larval tunnels: 0, no tunnels; 1, 1 to 2 tunnels < 0.5 cm; 2, 3 to 4 tunnels < 0.5 cm; 3, 5 to 6 tunnels 0.5 to 1.0 cm; 4, 7 to 8 tunnels 1.0 to 1.5 cm; 5, ≥ 9 tunnels > 1.5 cm. For each plot, the Pest Damage Index (PDI, %) was calculated as PDI = ($\sum_{i=0}^{5}n_{i}\times v_{i}$)/(N $\times v_{\max}$) × 100, where *n_i_* is the number of tubers at grade i, *v_i_* is the grade value (0 to 5), N = 50 is the total number of tubers evaluated, and v_max_= 5; the overall damage rate (%) was also calculated as (number of damaged tubers/total surveyed tubers) × 100%. In spring 2024, similar experiments were conducted in Longdong, Yunnan, China (25.26°N, 103.62°E) using two severely damaged susceptible and two lightly damaged cultivars, with three replicates each (12 plots total). Plot design and methods matched those of 2023, with larval counts recorded after emergence. Before performing ANOVA, the field survey data were tested for normality using the Shapiro–Wilk test and were found to meet the assumptions for parametric analysis. Differences among potato cultivars in *P. operculella* infestation and damage indices were evaluated using one-way ANOVA.

### Selection Preference of *P. operculella* on Different Potato Cultivars.

Four potato cultivars, namely Jingzhang1, Jingzhang4, G26, and G22 (all of them from Hebei Agricultural University), were selected. After harvest, smooth, intact, disease-free tubers were stored at 16 °C in the dark, sprouted, and transplanted into pots (15 cm × 10 cm, peat soil) in a greenhouse (16 h light at 22 ± 1 °C/8 h dark at 18 ± 1 °C, daily irrigation). 30-d-old seedlings were used in Y-tube dual-choice assays. For oviposition choice, pots were sealed with aluminum foil and placed in cages (56 × 56 × 60 cm), with plants in each corner. In no-choice assays, single-cultivar plants were placed in plastic cages, each with 10 female and 12 male moths; honey water was refreshed daily. Oviposition was recorded after 72 h using a dissecting microscope, with at least 10 replicates per test. In both assays, 2-d-old virgin adults were used (unfed before testing). Before performing ANOVA, the oviposition data were tested for normality using the Shapiro–Wilk test and met the assumptions for parametric analysis. The total oviposition counts of *P. operculella* on different plant cultivars were analyzed using one-way ANOVA to differentiate selection preferences among potato cultivars.

### Y-Tube Dual-Choice Assays.

For olfactometer tests, a Y-tube system (14 cm stem, two 10 cm arms at 60°, 15 mm ID) was used, with air filtered through activated charcoal and distilled water, and behavior recorded under IR light. Newly emerged females were paired with males before testing; mated females were collected into 10 mL centrifuge tubes during scotophase and assayed at 24 h and 48 h postmating (i.e., 2 to 3-d-old adults). Females entered via the stem and had 5 min to choose; nonresponders were excluded. Odor source positions were alternated every five females. The Y-tube was replaced with a clean unit after every 10 insects; after each use, the Y-tube was rinsed with ethanol and sterilized at 180 °C for 3 h before being reused. For full-plant tests, pots were covered with aluminum foil, and airflow (0.1 L/min) was adjusted to minimize soil volatiles (37). Each plant pair was tested with >50 females. Y-tube choices (responders only) were analyzed in R (v4.1.2) (two-tailed χ^2^ test vs. 50:50; α = 0.05).

### Insect Choice Assay with a Four-Arm Olfactometer.

A four-arm olfactometer (27) was used to assess behavioral responses of gravid female *P. operculella* to candidate plant volatiles. Based on published designs (38), we built an enlarged four-arm olfactometer for behavioral tests of the potato tuber moth. The system included an activated carbon column, water column, flow meter, odor vials, arena module (Teflon, octagonal, 30 cm long/26 cm short diagonal, 5 cm height, central groove 26 × 2 cm), and a vacuum pump, connected with Teflon tubing and operated in a darkroom. Behavioral assays were performed after sunset. Gravid females were acclimated in 5 mL centrifuge tubes in darkness for 2 h. Test compounds (10 μL, 1 mg/mL in paraffin oil) or paraffin oil control (10 μL) were applied to 1 × 2 cm filter paper in odor vials. This dose was selected based on preliminary range-finding assays and published moth olfactometer studies, as it reliably elicited behavioral responses without nonspecific effects due to overloading. The airflow was kept at 800 mL/min. After 5 min preconditioning, two insects were introduced into the arena. Infrared video recorded activity for 15 min. The olfactometer was rotated 90° after each trial; new filter papers were used, and the chamber was cleaned with 75% ethanol. Moths moving ≥10 cm in 5 min were considered valid; others were excluded. Residence time in each region was video-tracked, and odor preference was calculated as the ratio of residence time in the odor region to the total observation time. The residence time ratios between the odor and control (CK) regions were compared to determine whether insects showed a significant preference or avoidance. All datasets were first tested for normality using the Shapiro–Wilk test. Normally distributed data were analyzed using the independent-samples *t* test, while non-normally distributed data were analyzed using the Mann–Whitney *U* test. Differences were considered statistically significant at *P* < 0.05.

### VOCs Analysis by GC–MS.

Four selected potato cultivars (Jing1, Jing4, G22, and G26) were grown in a greenhouse, and foliar volatiles were analyzed for 30 d postplanting. Mature leaves from the midsection of each plant were excised, flash-frozen in liquid nitrogen, and stored at −80 °C. For analysis, frozen samples were ground in liquid nitrogen to keep the tissue at cryogenic temperatures and minimize volatilization during homogenization, and ~500 mg of powder was transferred to a 20 mL headspace vial (Agilent Technologies, Santa Clara, CA) containing a NaCl-saturated solution. Then, 20 μL of internal standard (3-hexanone, 50 μg/mL) was added. Volatiles were extracted using HS-SPME and analyzed by an Agilent 8890 GC coupled to a 7,000E triple quadrupole MS (GC–MS), equipped with a 30 m × 0.25 mm × 0.25 μm DB-5MS capillary column (5% phenyl-polymethylsiloxane). Compounds were identified using full-scan spectra with retention index confirmation and quantified using time-segmented SIM with compound-specific ions selected from a custom database (39). Metabolites were identified and quantified using a custom database (MetWare, Wuhan, China) and MassHunter Quantitative Analysis software (Agilent Technologies, Santa Clara, CA). Semiquantitative normalization used internal standards. Univariate analyses included hypothesis testing and fold-change (FC) analysis, and multivariate analyses were performed using OPLS-DA. Differential metabolites were selected by integrating VIP values from the OPLS-DA model (biological replicates ≥ 3) with univariate metrics (FC and *P* value/FDR; biological replicates ≥ 2). Metabolites were considered differential when VIP > 1 and FC ≥ 2 or FC ≤ 0.5, with FDR < 0.05.

### EAG Assay.

A total of 15 VOCs were selected for EAG based on metabolomics. Compounds were dissolved in liquid paraffin (100 mg/mL stock, 1 mg/mL working), stored at –20 °C. Mated 2 to 3-d-old adults were prepared under a stereomicroscope, antennae excised, and mounted with conductive gel (Sigma-Aldrich, St. Louis, MO) on EAG-Probe electrodes (Syntech, Kirchzarten, Germany). Stimulus (1 L/min humidified air, 0.3 s pulses) was controlled by CS-05 (Syntech, Kirchzarten, Germany). Signals were digitized with IDAC-4 and analyzed using EAG Pro v2.7 (Syntech, Kirchzarten, Germany). For each compound, ≥6 biological replicates were tested, with 1 min intervals. Before statistical comparison, all data were tested for normality using the Shapiro–Wilk test and for homogeneity of variances using Levene’s test. Normally distributed data with equal variances were analyzed using independent-samples *t* tests to compare male and female responses. When the data did not meet parametric assumptions, the Mann–Whitney *U* test was used instead.

### Tissue Expression Validation of OR Genes by RT-qPCR.

To validate tissue-specific expression, we selected 31 OR genes, including PopeOrco, from our previously generated antennal transcriptome; candidates were chosen for female antenna-biased expression in that dataset (28). Tissues collected included: 80 first-instar larvae, 80 heads of second- and third-instar, 60 heads of fourth-instar, and 60 late fourth-instar larvae; 120 antennae and 40 male genitalia from unmated males; and 120 antennae, 40 heads, 40 legs, and 40 ovipositors from 2 to 3-d-old females of two mating statuses. Each tissue type had three biological replicates. All samples were flash-frozen in liquid nitrogen and stored at −80 °C. Total RNA was extracted using TRIzol reagent (Ambion, Thermo Fisher Scientific, Waltham, MA), with purity/integrity assessed using a NanoDrop 2000 (Thermo Fisher Scientific, Waltham, MA) and a 2% agarose gel. First-strand cDNA was synthesized with 1 μg RNA using TransScript All-in-One SuperMix for PCR (TransGen Biotech, Beijing, China). Gene-specific primers were designed (Beacon Designer 8.14) and synthesized by Sangon Biotech (Shanghai, China). Elongation factor (EF) and ribosomal protein L13 (RPL13) were used as internal controls (40). Standard curves for qPCR were generated using twofold antennal cDNA dilutions; only primers with R^2^ > 0.98, 90 to 110% efficiency, and single-peak melting curves were used (Dataset S4). Chemosensory organ cDNA was diluted 5- to 10× for PCR. The qPCR mixture (15 μL) comprised 1 μL of template cDNA, 7.5 μL PerfectStart® Green qPCR SuperMix (TransGen Biotech, Beijing, China), 0.6 μL of forward/reverse primers, and 5.3 μL of nuclease-free water. Reactions were performed in 384-well plates on a QuantStudio™ 6 Flex system (Thermo Fisher Scientific, Applied Biosystems, Waltham, MA) using the following conditions: initial denaturation at 95 °C for 10 min, followed by 40 cycles of 95 °C for 5 s, 60 °C for 34 s, and 72 °C for 10 s. Three biological replicates per tissue were analyzed, each with four technical replicates. Negative controls (nuclease-free water instead of cDNA) were placed. Relative gene expression levels were calculated using the 2^−ΔΔCt^ method (41). Data analysis and visualization were performed using GraphPad Prism software (GraphPad Software, San Diego, CA). Normality was assessed (Shapiro–Wilk); normally distributed data were analyzed with Student’s *t* tests, otherwise with Mann–Whitney U tests (*P* < 0.05).

### Cloning and Functional Characterization of ORs.

Based on an expression survey, 17 OR genes, including PopeORco, were selected for functional assays, guided by high expression in postmating female antennae or by persistently female-biased antennal expression relative to males. Gene-specific primers were designed with Primer Premier 6.0 (Dataset S5). Using cDNA from female antennae, PCR was performed in 50 μL reactions: 25 μL 2 × TransStart® FastPfu Fly PCR SuperMix (TransGen Biotech, Beijing, China), 2 μL each primer, 2 μL cDNA, and nuclease-free water. PCR cycling: 95 °C for 5 min; 35 cycles of 95 °C for 20 s, 60 °C for 20 s, 72 °C for 2 min; final extension at 72 °C for 10 min. Products were ligated into pEASY®-Blunt vector (TransGen Biotech, Beijing, China) and sequenced by Sangon Biotech (Shanghai, China). For functional analysis, PopeORs and PopeOrco ORFs were subcloned into pGEMHE (Dataset S5). For cRNA synthesis with the mMESSAGE mMACHINE T7 Kit (Ambion, Thermo Fisher Scientific, Waltham, MA), pGEMHE vectors were linearized with NaeI. 20 nL PopeORs + PopeOrco cRNA (1,000 ng/μL) was microinjected into stage V/VI *X. laevis* oocytes (Drummond, Broomall, PA). Oocytes were incubated 2 to 6 d at 18 °C in Barth’s solution (96 mM NaCl, 2 mM KCl, 5 mM MgCl_2_ 0.6 mM CaCl_2_ 5 mM HEPES, pH 7.6, antibiotics, and sodium pyruvate). Two-electrode voltage-clamp recordings were performed at a holding potential of −80 mV using an OC-725C Oocyte Clamp (Warner Instruments, Hamden, CT) coupled to a Digidata 1550A interface and pCLAMP 10/Clampfit 10 (Molecular Devices, San Jose, CA). Stock solutions (1 M in DMSO) of all compounds were diluted with Ringer buffer. Oocytes were tested with plant volatiles identified from cultivars and other Solanaceae compounds reported to be active against *P. operculella*.

### CRISPR/Cas9-Mediated Knockout of PopeOR01 and PopeOR73 and Functional Validation.

To investigate the functional roles of PopeOR01 and PopeOR73, CRISPR/Cas9-mediated gene knockout was performed. For PopeOR01, two single-guide RNAs (sgRNAs) were designed to target exons 1 and 2, resulting in a deletion of approximately 533 bp. For PopeOR73, two sgRNAs were designed within exon 4, resulting in a deletion of approximately 406 bp. The primers used for sgRNA synthesis and genotyping are listed in Dataset S7. All sgRNAs were synthesized using the GeneArt^®^ Precision gRNA Synthesis Kit (Thermo Fisher Scientific, Waltham, MA). The sgRNAs and TrueCut™ Cas9 Protein v2 (Thermo Fisher Scientific, Waltham, MA) were mixed to final concentrations of 200 ng/μL (sgRNA) and 1,000 ng/μL (Cas9), respectively. The mixture was injected into newly fertilized eggs (preblastoderm stage) using an Eppendorf FemtoJet Ni4 Microinjector (Eppendorf, Germany), as previously described for Lepidoptera (42). After adult emergence, the middle legs were removed from individual moths, and genomic DNA was extracted using the EasyPure^®^ Marine Animal Genomic DNA Kit (TransGen Biotech, Beijing, China). The extracted DNA was used as a template for genotyping by PCR amplification with 2× EasyTaq^®^ PCR SuperMix (+dye) (TransGen Biotech, Beijing, China). Mutations were verified by PCR amplification and Sanger sequencing, and homozygous knockout lines (PopeOR01^−/−^ and PopeOR73^−/−^) were established for further analysis. Because our study focuses on cultivar-specific host selection and oviposition, which are primarily determined by postmating gravid females, we prioritized electrophysiological and behavioral assays in homozygous knockout females. Moreover, *PopeOR01* and *PopeOR73* were selected based on female-biased antennal expression and/or high expression in postmating female antennae; therefore, EAG recordings in mutant males were not conducted in this study. EAG recordings were performed using G3-generation adult moths from the homozygous knockout lines to evaluate antennal responses to the respective ligands, i.e., nerolidol for PopeOR01 and 3-carene for PopeOR73. Dose–response experiments were conducted at a series of concentrations (0.001, 0.01, 0.1, 1, 10, and 100 mg/mL) using the same procedures as for WT moths. Behavioral tests were carried out using G3-generation homozygous knockout female adults, with nerolidol, and β-ionone for PopeOR01^−/−^, and 3-carene and benzyl tiglate for PopeOR73^−/−^.

For the multiple-choice oviposition assay, pot soil surfaces were sealed with aluminum foil to prevent oviposition on the soil. Four potted plants (cultivars Jing1, Jing4, G22, and G26) were placed simultaneously in mesh cages (56 × 56 × 60 cm), with one plant positioned at each corner. For the no-choice assay, potted plants of a single cultivar were placed in plastic cages, and each cage was supplied with 10 females and 12 males. Honey water was provided and refreshed daily. In both assays, 2-d-old virgin adults from the homozygous knockout lines were used and were kept unfed prior to testing. After 72 h, eggs laid on each plant were counted under a dissecting microscope. Each treatment was replicated at least 10×. Prior to ANOVA, data were tested for normality and met the assumptions required for parametric analysis. Oviposition counts were analyzed by one-way ANOVA followed by Tukey’s post hoc test to evaluate cultivar-specific oviposition preferences.

## Supplementary Material

Appendix 01 (PDF)

Dataset S01 (XLSX)

Dataset S02 (XLSX)

Dataset S03 (XLSX)

Dataset S04 (XLSX)

Dataset S05 (XLSX)

Dataset S06 (XLSX)

Dataset S07 (XLSX)

Dataset S08 (XLSX)

Dataset S09 (XLSX)

## Data Availability

All study data are included in the article and/or supporting information.
