# Supplementary material for: Semiochemicals and odorant receptors underlying potato cultivar susceptibility and resistance to potato tuber moth
Source: Proc Natl Acad Sci U S A. 2026 Apr 6;123(15):e2537754123. doi: 10.1073/pnas.2537754123 (PMC13079372; doi:10.1073/pnas.2537754123)
Supplement: Supplementary file 1 — Appendix 01 (PDF) [file pnas.2537754123.sapp.pdf]

PNAS

Supporting Information for

Semiochemicals and odorant receptors underlying potato cultivar susceptibility and resistance to potato tuber moth

Ruipeng Chen[1], Wangtao Hu[1], Qin Hu[1], JunJie Yan[1], Jiao Yin[1], Fathiya M. Khamis[2,3], Yulin Gao[1,4]*, Walter S Leal[5]*.

[1]State Key Laboratory for Biology of Plant Diseases and Insect Pests, Institute of Plant Protection, Chinese Academy of Agricultural Sciences, Beijing 100193, China

[2]International Center of Insect Physiology and Ecology (ICIPE), Nairobi P.O. Box 30772-00100, Kenya

[3]Department of Zoology and Entomology, University of Pretoria, Hatfield 0028, South Africa

[4]Institute of Bast Fiber Crops & Center of Southern Economic Crops, Chinese Academy of Agricultural Sciences, Changsha 410205, China

[5]Department of Molecular and Cellular Biology, University of California-Davis, CA 95616, USA

*Correspondence: gaoyulin@caas.cn, wsleal@ucdavis.edu

This PDF file includes:

Supporting Text
Supporting Material and Methods
Supporting Figures S1 to S14

Supporting Text

GenBank accession numbers

PopeOR01 (PX745330), *PopeOR04* (PX745331), *PopeOR05 (PX745332), PopeOR15* (PX745333), *PopeOR16 (*PX745334), *PopeOR31* (PX745335), *PopeOR37* (PX745336), *PopeOR45* (PX745337), *PopeOR47* (PX745338), *PopeOR48* (PX745339), *PopeOR66* (PX745340), *PopeOR72* (PX745341), *PopeOR73* (PX745342), *PopeOR75* (PX745343), *PopeOR87* (PX745344), *PopeOR88* (PX745345), and *PopeOrco* (PX745346).

CAS number of studies compounds

Camphene (CAS #79-92-5), ethyl cinnamate, benzyl tiglate (CAS #37526-88-8), nerolidol (CAS 7212-44-4), 3-carene (CAS #13466-78-9), 2-methoxy-3-methylpyrazine (CAS #2847-30-5), phenethyl butyrate (CAS # 3460-44-4), 2,4-octadienal (CAS #30361-28-5), 2,4-undecadienal (CAS 30361-29-6), fenchol (CAS #1632-73-1), ethyl mandelate (CAS #774-40-3), β-ionone (CAS #79-77-6), 2-methylbutyl isovalerate (CAS #2445-77-4), and (*Z*)-3-hexenol (CAS #928-96-1).

Molecular recognition of plant volatiles by PopeORs

AlphaFold3 was used to predict the three-dimensional structures of OR–Orco complexes for PopeOR01, PopeOR15, and PopeOR73 (Fig. S11A–G). Structural comparison revealed that the predicted PopeORco model closely matched the ApisORco cryo-EM structure (PDB: 8Z9Z), including the presence of two antiparallel β-strands in the S3–S4 loop, with an α-carbon RMSD of 0.807 Å across 354 aligned Cα atoms (Fig. S11D).

The predicted models of PopeOR01, PopeOR15, and PopeOR73 showed overall structural homology to ApisOR5 but also displayed notable differences. Within the predicted membrane boundaries, PopeOR01 exhibited an overall RMSD of 2.12 Å across 277 aligned Cα atoms relative to ApisOR5 (Fig. S11E), with per–transmembrane helix RMSDs reported in Dataset S8. Similarly, PopeOR15 showed an overall RMSD of 2.22 Å across 273 aligned Cα atoms (Fig. S11F; Dataset S8), and PopeOR73 displayed an overall RMSD of 2.09 Å across 261 aligned Cα atoms compared with ApisOR5 (Fig. S11G; Dataset S8).

To investigate the molecular basis of receptor-ligand interactions observed in functional assays, we performed molecular docking followed by molecular dynamics (MD) simulations on the three most behaviorally and electrophysiologically active receptor-ligand pairs: PopeOR73 with 3-carene, PopeOR01 with nerolidol, and PopeOR15 with benzyl tiglate. Docking and simulations were conducted using the DeepSite-predicted binding cavity for each receptor. Together with our dose–response analyses (EC50; Dataset S9), these structural models provide a quantitative and mechanistic framework for interpreting receptor sensitivity and tuning specificity, and for inferring potential responsiveness to additional, as yet uncharacterized VOCs.

Docking of 3-carene into the predicted binding pocket of PopeOR73 yielded a minimum binding energy of –7.5 kcal/mol, the lowest among 121 screened volatiles (Fig. S12A,B; Dataset S9a), indicating strong ligand-receptor compatibility. In the top-ranked pose, 3-carene was stabilized primarily through nonpolar interactions within a hydrophobic pocket. MD simulations (Fig. S12C) showed that the protein Cα RMSD stabilized at 1.5–1.8 Å, while the ligand RMSD remained at 0.7-1.0 Å, consistent with a stable complex.

Per-residue MMGBSA energy decomposition identified PHE386 and TYR157 as the major contributors to binding, with GLN383, LEU87, ILE382, VAL205, and THR208 providing additional stabilization. These results highlight the importance of aromatic and hydrophobic interactions in 3-carene recognition (Fig. S12D; Dataset S9b). The compact, highly hydrophobic nature of the PopeOR73 pocket is consistent with its behavioral and electrophysiological role in detecting low-polarity monoterpene hydrocarbons and suggests that PopeOR73 may also respond to other structurally related monoterpenes beyond 3-carene.

Similarly, nerolidol docked into the DeepSite-predicted cavity of PopeOR01 with a minimum binding energy of –7.8 kcal/mol (Fig. S13A). The top-ranked pose revealed a hydrogen bond between the carbonyl oxygen of nerolidol and GLN314, anchoring the ligand within the hydrophobic pocket (Fig. S13B). Comparative docking against 121 volatiles confirmed nerolidol as the lowest-energy binder (Dataset S9c), consistent with electrophysiological responses. MMGBSA per-residue decomposition showed that TYR46, GLY65, PHE70, LEU73, and GLN314 contributed most strongly to ligand stabilization (each < -1.0 kcal/mol; Fig. S13C; Dataset S9d). During 300 ns MD simulations, the protein backbone RMSD remained at ~2.0 Å and the ligand RMSD at ~2.2 Å (Fig. S13D), indicating a stable PopeOR01–nerolidol complex. Notably, the ‘polar anchor (GLN314) + hydrophobic enclosure’ binding logic provides a structural basis for the high sensitivity of PopeOR01 (low EC50; Dataset S9d) and implies potential responsiveness to additional sesquiterpene alcohols or related terpenoid derivatives with similar elongated hydrophobic scaffolds and polar functional groups.

Docking of benzyl tiglate into the predicted binding cavity of PopeOR15 resulted in a top binding energy of –8.7 kcal/mol (Fig. S14B), the lowest among all screened ligands (Dataset S9e). The selected pose (Model 1) featured a hydrogen bond between the ligand’s carbonyl oxygen and TYR162 (Fig. S14A). MD simulations over 300 ns showed that the protein backbone Cα RMSD stabilized at 1.6-1.8 Å, while ligand RMSD remained between 1.5 and 2.1 Å, supporting a stable binding mode (Fig. S14C). MMGBSA energy decomposition identified PHE87, CYS162, TYR166, and PHE169 as the primary contributors to binding (each < -1.0 kcal/mol), with LEU141, THR223, and VAL226 also contributing significantly (Fig. S14D; Dataset S9f). Consistent with its dose–response profile (EC50; Dataset S9f), these aromatic/hydrophobic contacts, together with a defined polar anchoring interaction, suggest that PopeOR15 may preferentially recognize aromatic ester VOCs and potentially respond to additional, as yet untested esters sharing a similar aromatic scaffold and the positioning of carbonyl functionality.

Supporting Material and Methods

Computational simulation of ORs binding to plant volatiles.

3D models of three P. operculella odorant receptors (ORs) were generated using AlphaFold3, and Orco-OR complex models were built at a 3:1 ratio (1, 2). Individual OR structures were isolated in PyMOL (v3.1, Schrödinger, New York, NY, USA). Ligand files were batch-downloaded from PubChem using a Python script and converted via Open Babel. OR structures were prepared by removing water, adding polar hydrogens, and assigning Gasteiger charges using MGLTools (v1.5.7) (72). Grid box coordinates were set using DeepSite (https://open.playmolecule.org/tools/deepsite) (3). Molecular docking

was performed with AutoDock Vina (v1.2) (4), and results were visualized in PyMOL (v3.1, Schrödinger, New York, NY, USA). For molecular dynamics (MD) simulations, nerolidol-PopeOR01, benzyl tiglate-PopeOR15, and 3-carene-PopeOR73 complexes were assembled using docked poses. Proteins and ligands were prepared in Maestro (v2024-1, Schrödinger, New York, NY, USA), including pH adjustment, addition of H atoms, bond correction, and minimization. Membrane environments were generated using CHARMM-GUI (5) and oriented using OPM (6). Each system (protein, ligand, 200 POPC lipids, ~20,000 waters) was supplemented with 0.15 M KCl (1). MD was performed in Amber25 (7) using GAFF (8) for ligands, lipid21 for POPC, ff14SB (9) for protein, and TIP3P for water. Production runs were conducted at 298 K, with three independent 300 ns simulations per system (1). The particle mesh Ewald (PME) algorithm (10) was used for long-range electrostatics, with a 2 fs time step and periodic boundary conditions.

Figures

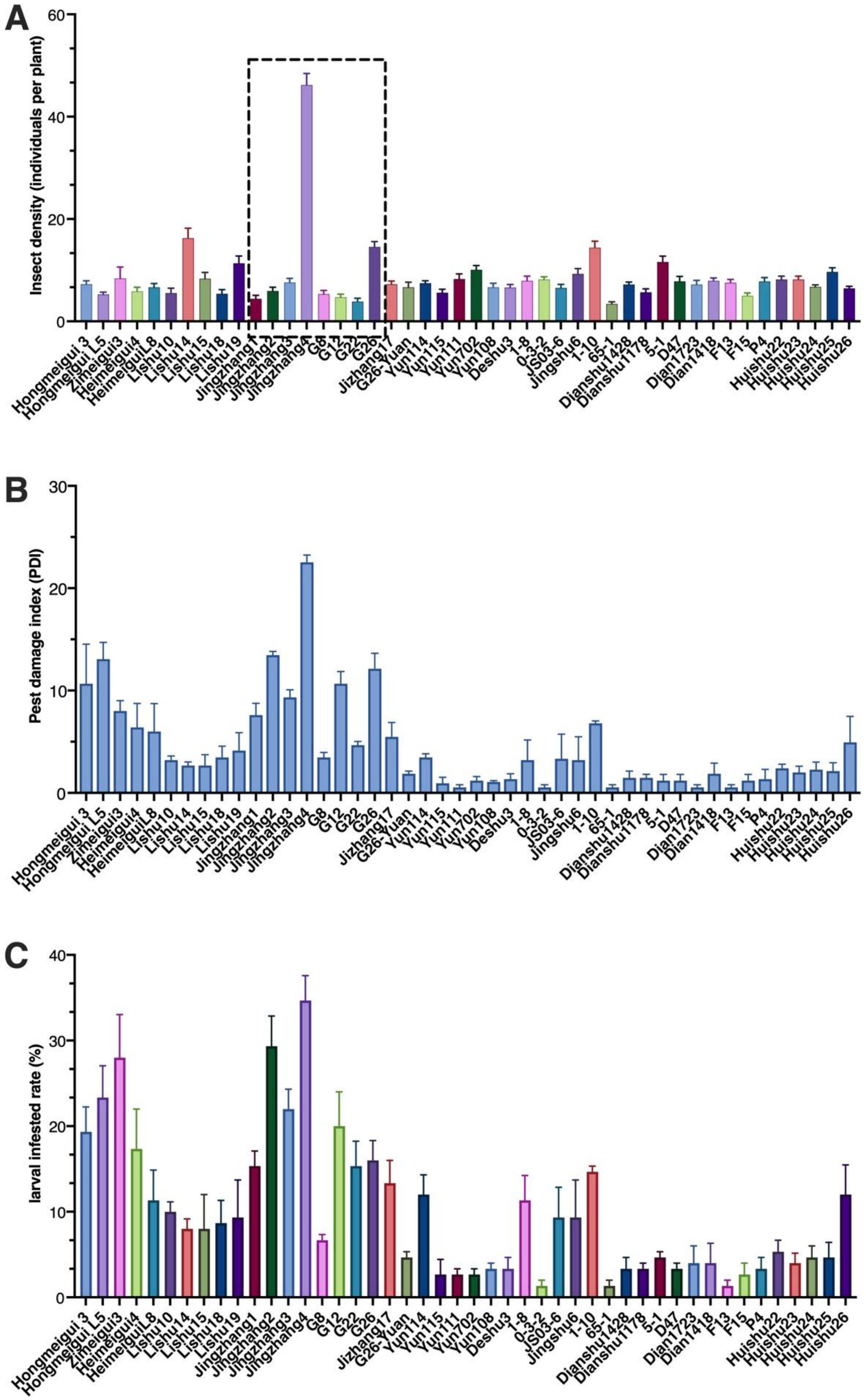
A
Insect density (individuals per plant)
60
40
20
0
B
Pest damage index (PDI)
30
20
10
0
C
larval infested rate (%)
40
30
20
10
0
Hongmeigui 3
Hongmeigui L5
Zimeigui3
Heimeigui4
HeimeiguiL8
Lishu10
Lishu14
Lishu15
Lishu18
Lishu19
Jingzhang1
Jingzhang2
Jingzhang3
Jingzhang4
G8
G12
G22
G26
Jizhang17
G26-Yuan
Yun114
Yun115
Yun111
Yun702
Yun108
Deshu3
1-8
0-3-2
JS03-6
Jingshu6
1-10
65-1
Dianshu1428
Dianshu1178
5-1
D47
Dian1723
Dian1418
F13
F15
P4
Huishu22
Huishu23
Huishu24
Huishu25
Huishu26

Figure S1. Field survey of *P. operculella* larval abundance across potato cultivars. (A) Mean larval density (insects per plant) recorded on each cultivar at the seedling stage under natural infestation. Bars represent mean ± SEM from three replicate plots. The dashed box highlights the eight cultivars selected for subsequent studies. (B) Larval infestation rate (%) of harvested tubers for each cultivar, representing belowground damage (mean ± SEM, *N* = 3 plots per cultivar). (C) Pest damage index (PDI) of tubers at harvest, assessed for each cultivar using the 0–5 damage scale described in Methods (mean ± SEM, *N* = 3 plots per cultivar).

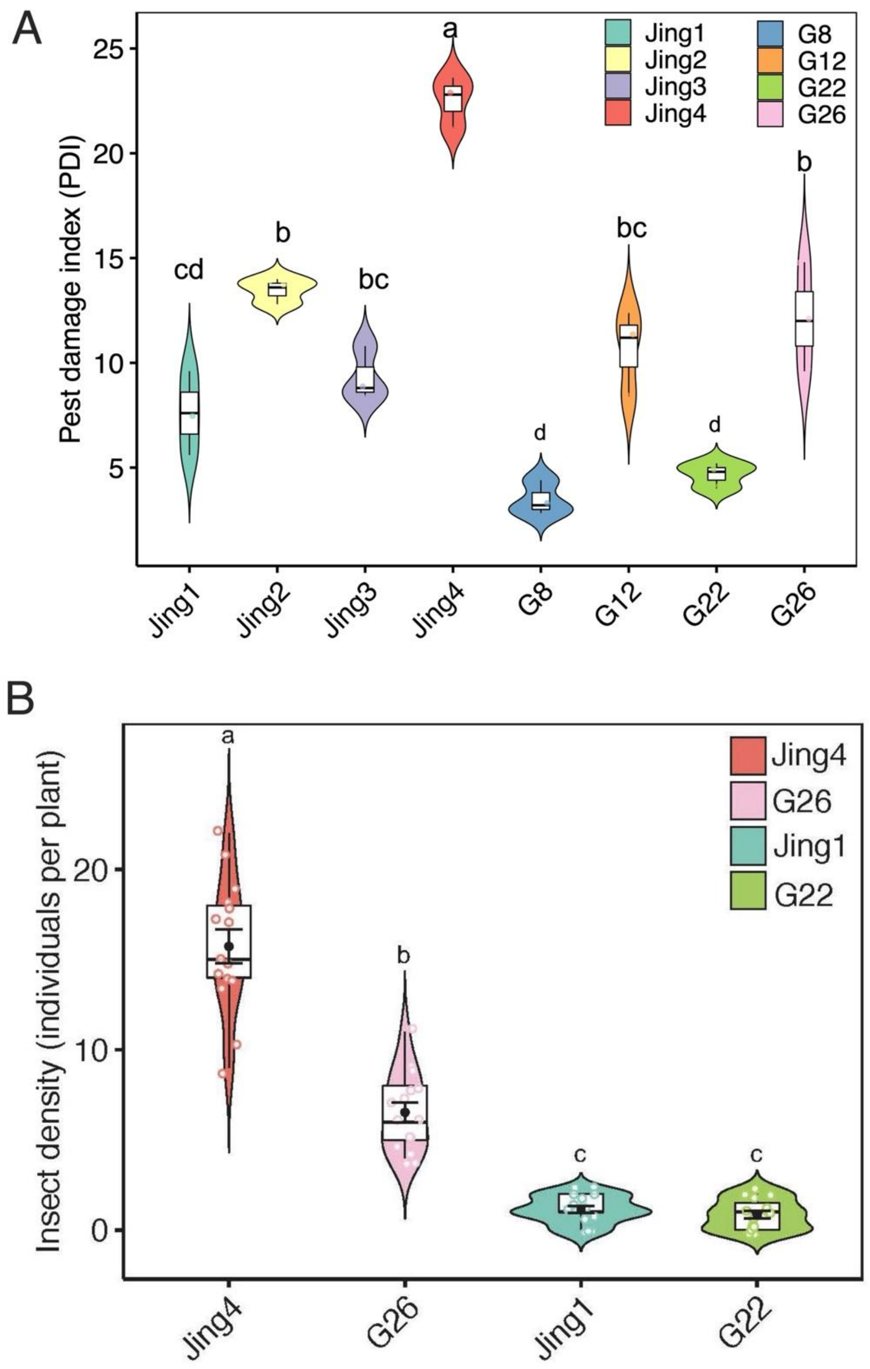

Figure S2. Pest damage and insect density survey in two different field studies. (A) Pest damage caused by the potato tuber moth in eight cultivars (2023 studies). (B) Field validation of *Phthorimaea operculella* infestation on four focal potato cultivars in 2024. Mean numbers of *P. operculella* larvae per plant were recorded for cultivars Jing4, G26, Jing1 and G22 at seedling emergence under natural infestation. Violin plots show the distribution of insect density (individuals per plant) across three replicate plots per cultivar; the internal boxplots indicate the median and interquartile range, and black dots denote the mean. Different letters above violin plots indicate significant differences among cultivars (one-way ANOVA followed by Tukey's HSD, $P < 0.05$).

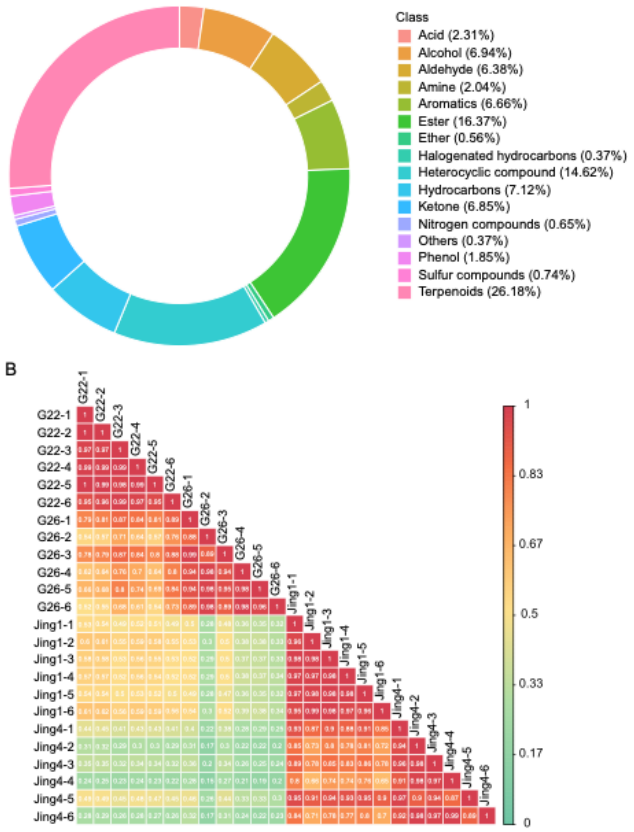

Figure S3. Overview of volatile organic compound (VOC) classes and profile reproducibility across four potato cultivars. (A) Ring chart showing the chemical classification of 1,081 VOCs detected in leaf headspace of Jing4, G26, Jing1 and G22 by GC–MS. Segments indicate the proportion (%) contributed by each class (e.g. terpenoids, esters, heterocyclic compounds, hydrocarbons, aldehydes, ketones, alcohols, etc.). (B) Pearson correlation matrix of VOC profiles from six biological replicates per cultivar (G22, G26, Jing1, Jing4). Each cell shows the correlation coefficient (r) between a pair of samples; color intensity represents correlation strength from low (green) to high (red), illustrating high within-cultivar reproducibility and clear between-cultivar differences.

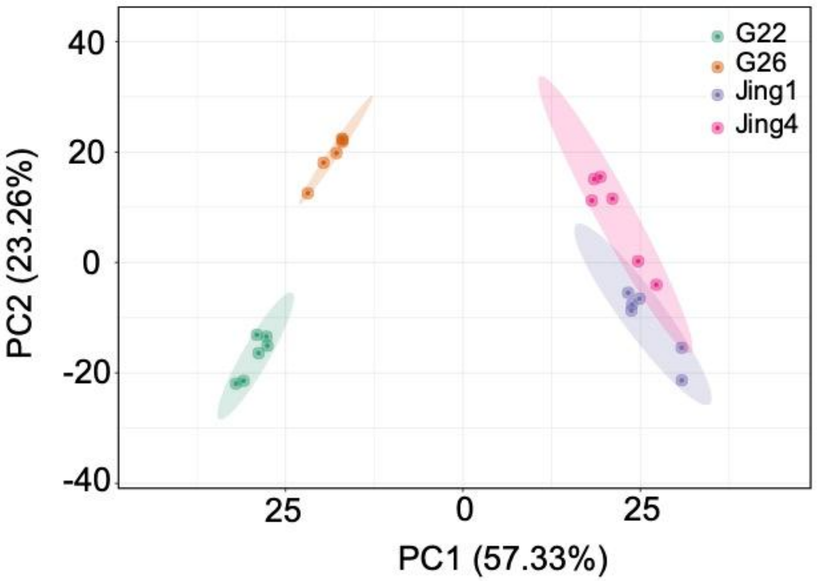

Figure S4. Principal component analysis (PCA) of leaf volatile profiles from four cultivars (Jing4, G26, Jing1, and G22). Each point represents one biological replicate; ellipses indicate 95% confidence regions. PC1 and PC2 explain 57.33% and 23.26% of the total variance, respectively.

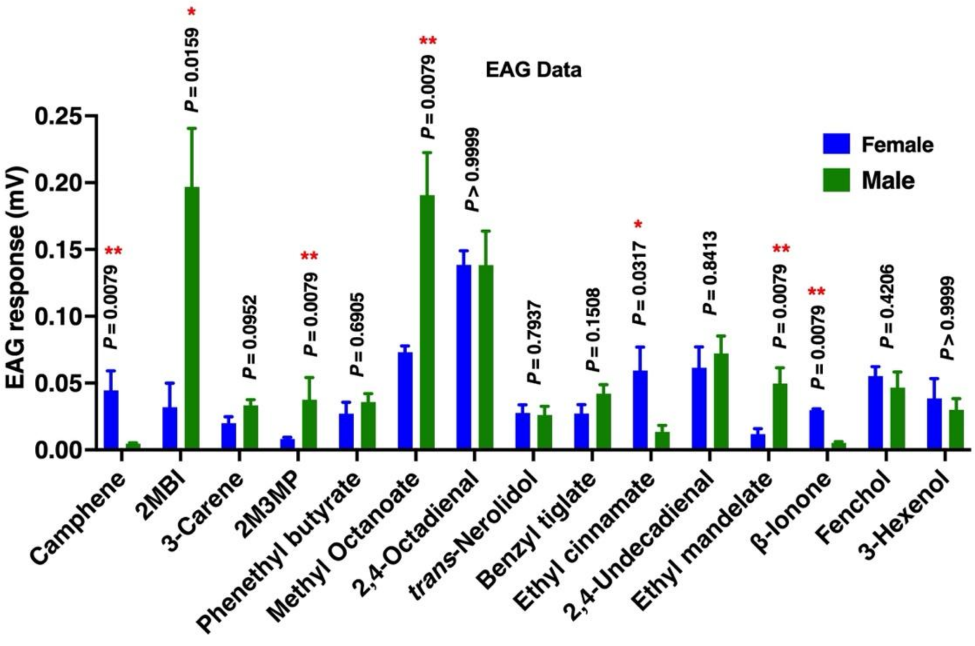

Figure S5. EAG responses (mean ± SE) of female and male antennae to the 15 VOCs. Bars show means, and open symbols represent individual replicates. Asterisks above bars indicate significant sex differences after the Mann–Whitney U test (**P* < 0.05, ***P* < 0.01, ****P* < 0.001). For brevity, 2-methylbutyl isovalerate and 2-methyl-3-methylpyrazine are abbreviated as 2MBI and 2M3MP, respectively, in this figure.

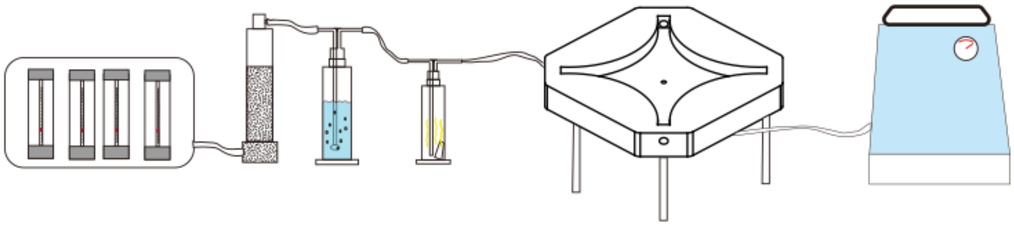

Figure S6. Schematic diagram of the four-arm olfactometer used for behavioral assays. Airflow was first divided into four streams using flowmeters, then sequentially filtered and humidified. Each stream passed through an odor vial containing either a test compound (dissolved in paraffin oil and applied to filter paper) or a solvent control and was delivered to one of the four arms of the olfactometer arena. A vacuum pump continuously drew air from the central outlet to maintain stable laminar airflow and prevent odor cross-contamination among arms.

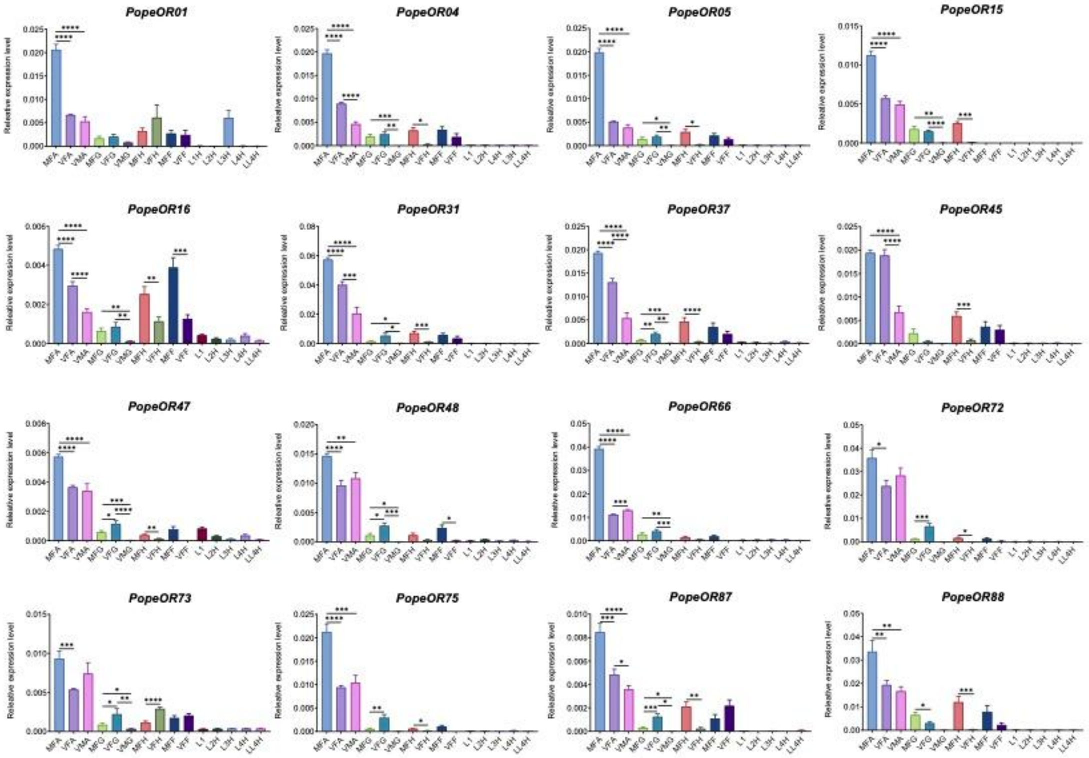

Figure S7. Tissue- and sex-specific expression profiles of selected *P. operculella* odorant receptor genes upregulated in mated females. Relative expression levels of 16 odorant receptor (*PopeORs*) genes across different tissues and developmental stages, measured by qRT-PCR. Samples include male antennae (VMA), male genitalia (VMG), female antennae (Virgin: VFA, Mated: MFA), Female legs (Virgin: VFF, Mated: MFF), ovipositor (Virgin: VFG, Mated: MFG), Female head (Virgin: VFH, Mated: MFH), and larval tissue (L1~LL4H). Error bars represent mean ± SEM. Asterisks indicate significant sex differences after a Shapiro–Wilk normality test: Student's t-test when normal, otherwise Mann–Whitney U (**P* < 0.05, ***P* < 0.01, ****P* < 0.001, *****P* < 0.0001).

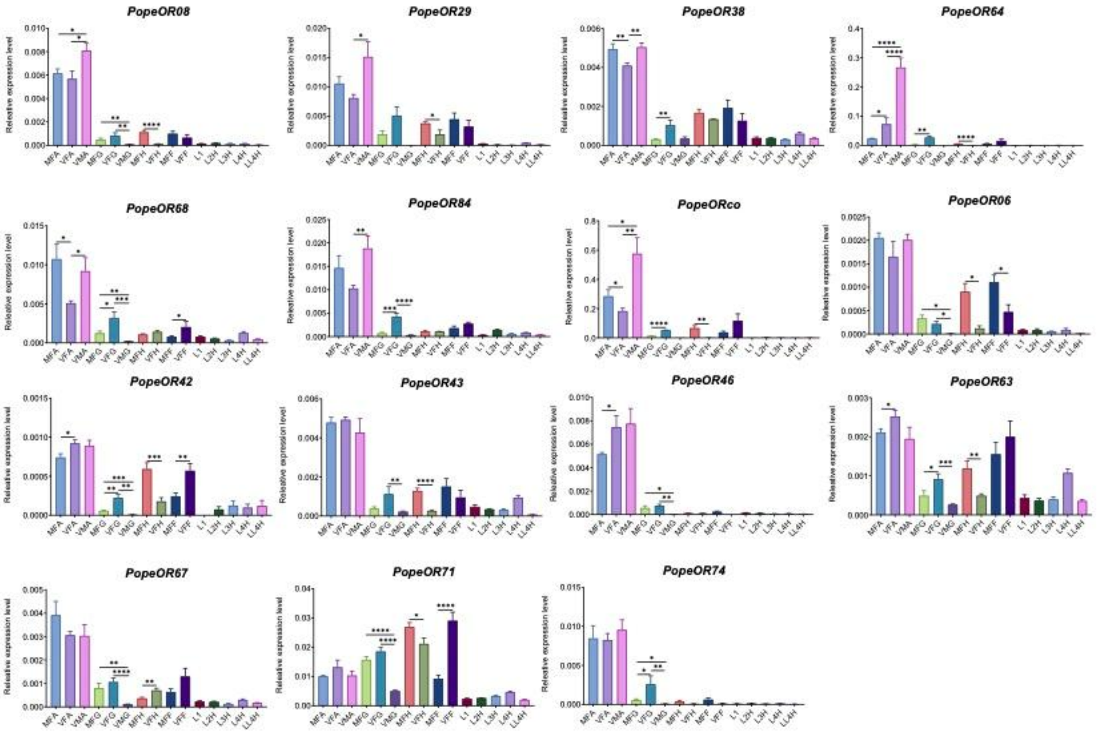

Figure S8. Tissue- and sex-specific expression profiles of selected *P. operculella* odorant receptor genes. Relative expression levels of 15 odorant receptor (*PopeORs*) genes across different tissues and developmental stages, measured by qRT-PCR. Samples include male antennae (VMA), male genitalia (VMG), female antennae (Virgin: VFA, Mated: MFA), Female legs (Virgin: VFF, Mated: MFF), ovipositor (Virgin: VFG, Mated: MFG), Female head (Virgin: VFH, Mated: MFH), and larval tissue (L1~LL4H). Error bars represent mean ± SEM. Asterisks indicate significant sex differences after a Shapiro–Wilk normality test: Student's t-test when normal, otherwise Mann–Whitney U (*$P < 0.05$, **$P < 0.01$, ***$P < 0.001$, ****$P < 0.0001$).

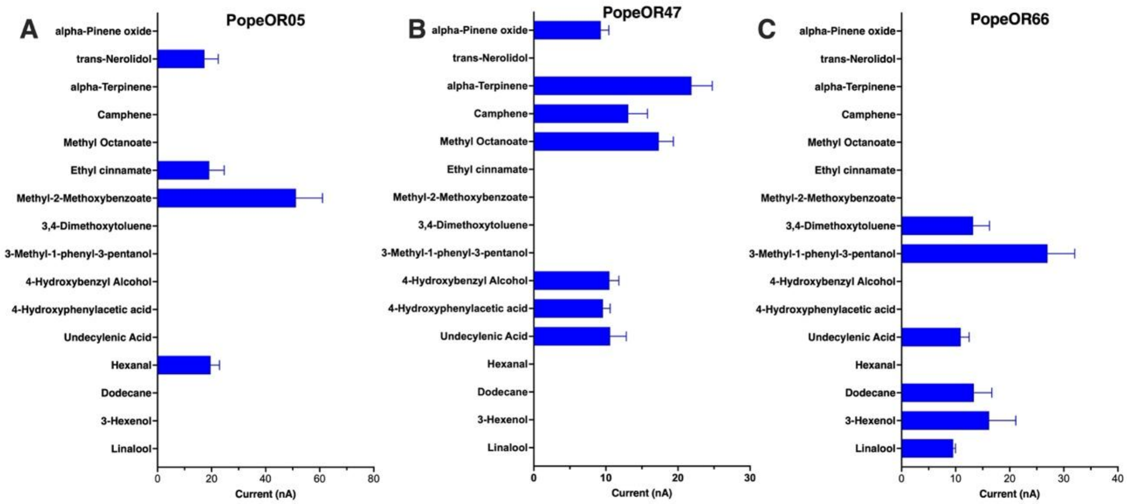

Figure S9. De-orphanization of promiscuous *P. operculella* odorant receptors. Quantification of current elicited by odorant receptors co-expressed with PopeOrco in *Xenopus* oocytes. (A) PopeOR5, (B) PopeOR47, and PopeOR66. Bars represent mean ± SEM from three replicates.

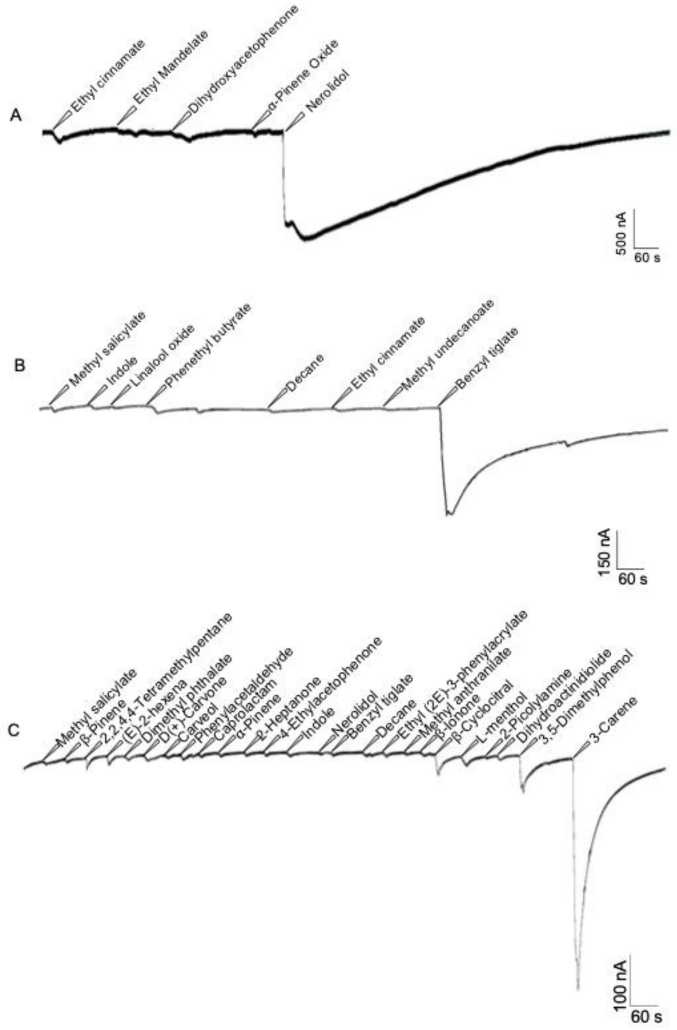

Figure S10. Representative TEVC traces illustrating activation of PopeOR01, PopeOR15 and PopeOR73 by plant volatile compounds. (A) Currents recorded from PopeOR01/PopeOrco-expressing oocytes challenged with ethyl cinnamate, ethyl mandelate, dihydroxyacetophenone, α-pinene oxide and nerolidol. (B) Trace obtained from an oocyte co-expressing PopeOR15 and PopeOrco and stimulated with methyl salicylate, indole, linalool oxide, phenethyl butyrate, decane, ethyl cinnamate, methyl undecanoate and benzyl tiglate. (C) Currents elicited by terpenoids, aromatic and aliphatic compounds from an oocyte co-expressing PopeOR73 and PopeOrco.

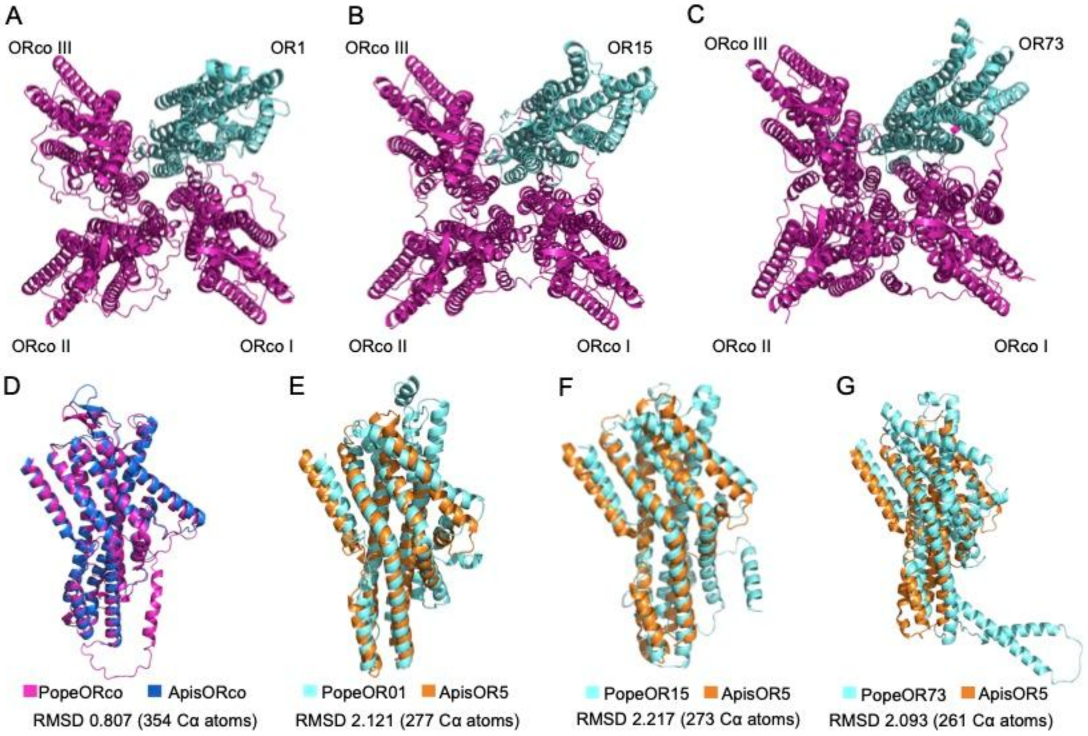

Figure S11. AlphaFold3-predicted quaternary and tertiary structures of PopeORco and its complexes with PopeOR01, PopeOR15, and PopeOR73 **(**A-C) Predicted quaternary structures of PopeORco in complex with PopeOR01, PopeOR15, and PopeOR73, respectively, based on AlphaFold3 modeling. The PopeORco homomeric trimer is shown in purple; individual PopeORs are shown in cyan. (D) Structural alignment of the predicted PopeORco model with the cryo-EM structure of ApisORco (PDB: 8Z9Z). The RMSD value (0.807 Å over 354 Cα atoms) indicates strong structural conservation. (E-G) Predicted PopeOR01, PopeOR15, and PopeOR73 structures aligned with ApisOR5 (PDB: 8Z9Z) to assess structural similarity. RMSD values are shown for each comparison: 2.12 Å (277 atoms) for OR01, 2.22 Å (273 atoms) for OR15, and 2.09 Å (261 atoms) for OR73, indicating conserved transmembrane domain architecture despite some local conformational differences.

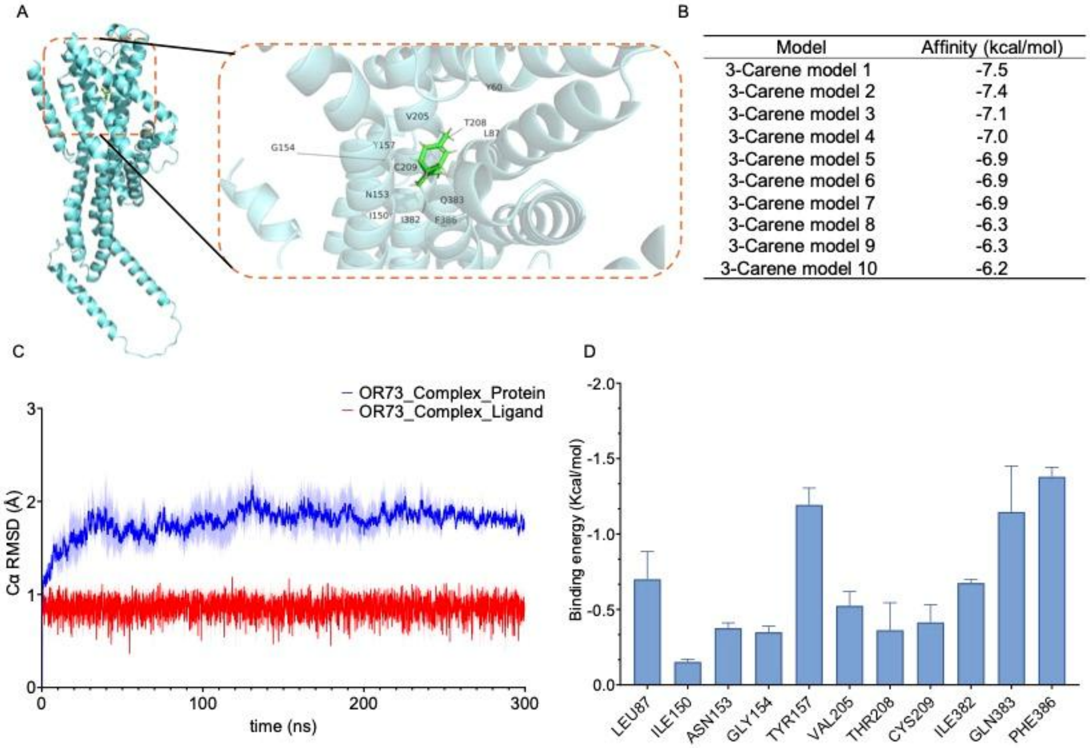

Model	Affinity (kcal/mol)
3-Carene model 1	-7.5
3-Carene model 2	-7.4
3-Carene model 3	-7.1
3-Carene model 4	-7.0
3-Carene model 5	-6.9
3-Carene model 6	-6.9
3-Carene model 7	-6.9
3-Carene model 8	-6.3
3-Carene model 9	-6.3
3-Carene model 10	-6.2

Figure S12. Molecular docking and MD simulation of 3-carene binding to PopeOR73. (A) Predicted 3D structure of PopeOR73, highlighting the transmembrane ligand-binding pocket. Zoomed-in view of the binding site showing the docking conformation of 3-carene (green). Residues such as V205, Y157, and T208 participate in hydrophobic interactions that stabilize the ligand. (B) Summary of binding affinities (kcal/mol) for the top 10 docking models, with the strongest pose yielding –7.5 kcal/mol. (C) RMSD analysis of the PopeOR73–3-carene complex during three independent 300-ns molecular dynamics simulations. Curves show the mean Cα RMSD of the protein (blue) and heavy-atom RMSD of the ligand (red), with shaded areas indicating ± SEM across the three replicates. (D) Per-residue MMGBSA binding energy decomposition for 3-carene in PopeOR73, reported as the mean ± SEM across the three simulations, identifying key contributing residues.

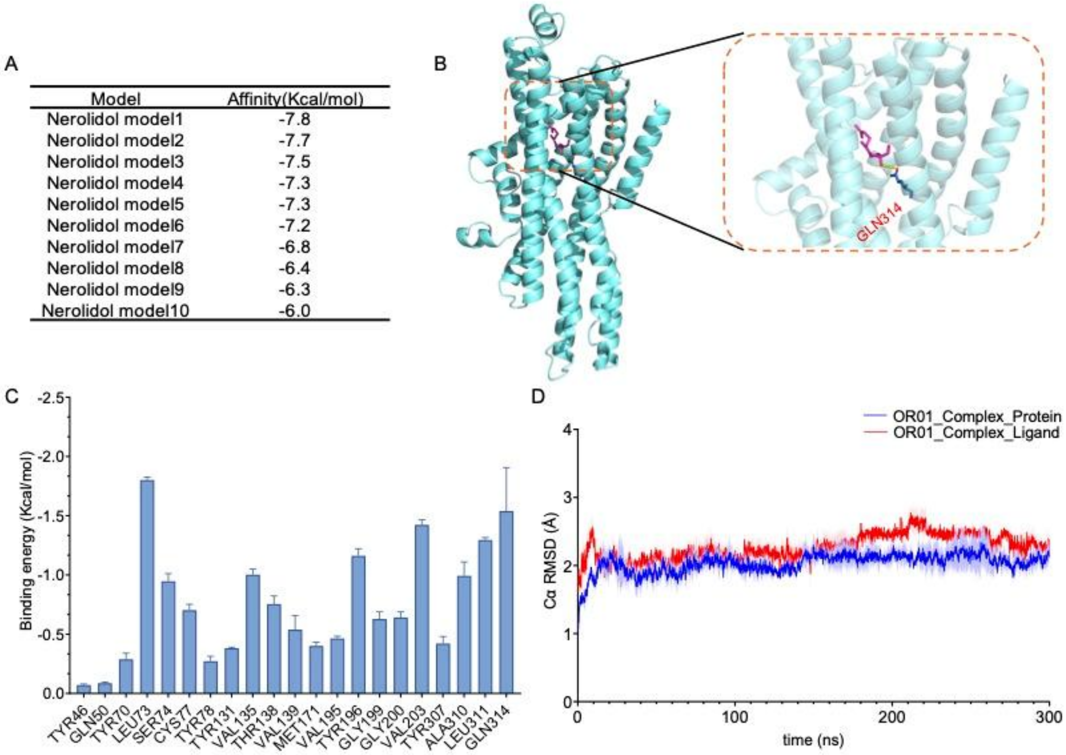

Figure S13. Molecular docking and MD simulation of nerolidol binding to PopeOR01. (A) Binding affinity (kcal/mol) of nerolidol across ten docking models into the predicted binding cavity of PopeOR01, with the most favorable model yielding –7.8 kcal/mol. (B) Predicted binding mode of nerolidol within the PopeOR01 transmembrane domain. The right panel shows a close-up view of the binding pocket, highlighting nerolidol in magenta and the key interacting residue, Gln314, in blue, which forms a hydrogen bond with the carbonyl oxygen of the ligand. (C) Per-residue MMGBSA binding energy decomposition for nerolidol in PopeOR01, reported as the mean ± SEM across three independent 300-ns molecular dynamics simulations, identifying key contributing residues. (D) RMSD analysis of the PopeOR01–nerolidol complex during three independent 300-ns molecular dynamics simulations. Curves show the mean Cα RMSD of the protein (blue) and heavy-atom RMSD of the ligand (red), with shaded areas indicating ± SEM across the three replicates.

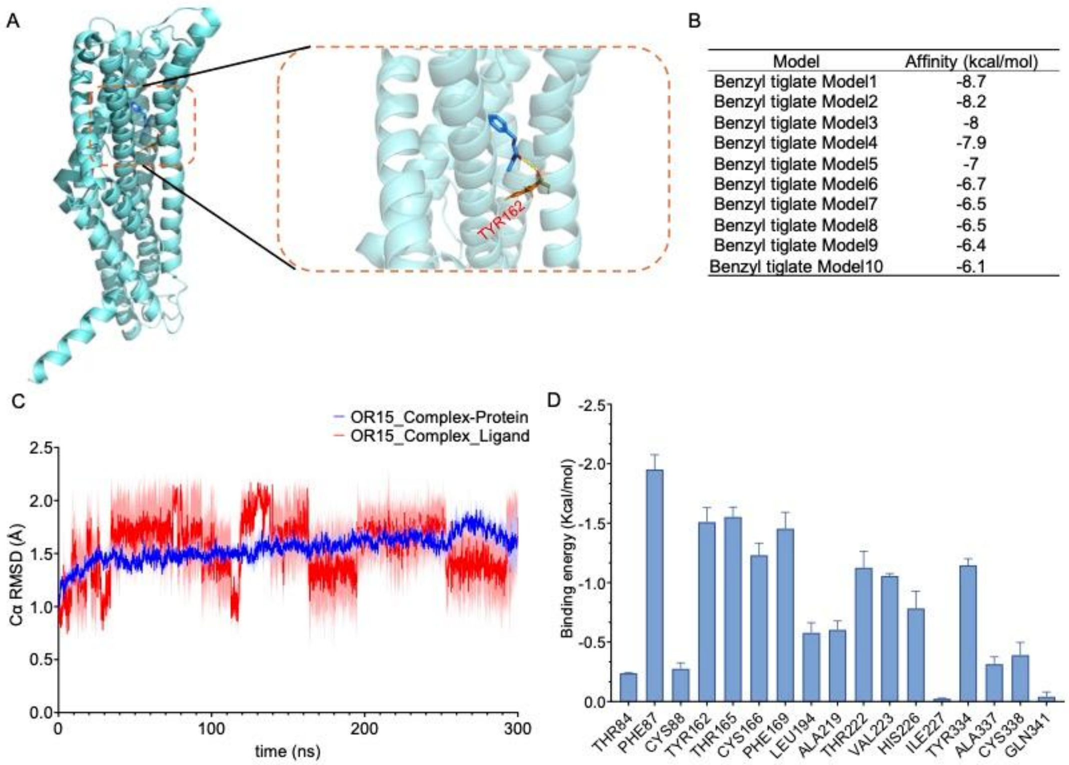

Figure S14. Molecular docking and MD simulation of benzyl tiglate binding to PopeOR15. (A) Predicted 3D structure highlighting the ligand-binding domain. Close-up view of the binding pocket showing benzyl tiglate (blue) docked into the predicted active site of PopeOR15. A stabilizing hydrogen bond is formed between the carbonyl group of the ligand and the side chain of Tyr162 (red), suggesting a key residue involved in ligand specificity. (B) Docking scores for the top 10 models are summarized in the table, with binding affinities ranging from –8.7 to –6.1 kcal/mol. (C) RMSD analysis of the PopeOR15–benzyl tiglate complex during three independent 300-ns molecular dynamics simulations. Curves show the mean Cα RMSD of the protein (blue) and heavy-atom RMSD of the ligand (red), with shaded areas indicating ± SEM across the three replicates. (D) Per-residue MMGBSA binding energy decomposition for benzyl tiglate in PopeOR15, reported as the mean ± SEM across the three simulations, identifying key contributing residues.

References

1. Y. Wang *et al.*, Structural basis for odorant recognition of the insect odorant receptor OR-Orco heterocomplex. *Science* **384**, 1453-1460 (2024).
2. J. Zhao, A. Q. Chen, J. Ryu, J. Del Mármol, Structural basis of odor sensing by insect heteromeric odorant receptors. *Science* **384**, 1460-1467 (2024).
3. M. Torrens-Fontanals, P. Tourlas, S. Doerr, G. De Fabritiis, PlayMolecule Viewer: A toolkit for the visualization of molecules and other data. *J. Chem. Inf. Model.* **64**, 584-589 (2024).
4. O. Trott, A. J. Olson, AutoDock Vina: improving the speed and accuracy of docking with a new scoring function, efficient optimization, and multithreading. *J. Comput. Chem.* **31**, 455-461 (2010).
5. S. Jo, T. Kim, V. G. Iyer, W. Im, CHARMM-GUI: A web-based graphical user interface for CHARMM. *J. Comput. Chem.* **29**, 1859-1865 (2008).
6. M. A. Lomize, I. D. Pogozheva, H. Joo, H. I. Mosberg, A. L. Lomize, OPM database and PPM web server: resources for positioning of proteins in membranes. *Nucleic Acids Research* **40**, D370-D376 (2011).
7. D. A. Case *et al.*, AmberTools. *J. Chem. Inf. Model.* **63**, 6183-6191 (2023).
8. J. Wang, R. M. Wolf, J. W. Caldwell, P. A. Kollman, D. A. Case, Development and testing of a general amber force field. *J. Comput. Chem.* **25**, 1157-1174 (2004).
9. J. A. Maier *et al.*, ff14SB: Improving the accuracy of protein side chain and backbone parameters from ff99SB. *J. Chem. Theory Comput.* **11**, 3696-3713 (2015).
10. T. Darden, D. York, L. Pedersen, Particle mesh Ewald: An N·log(N) method for Ewald sums in large systems. *J. Chem. Phys.* **98**, 10089-10092 (1993).

Dataset S1 Names and sources of the studied cultivars.

Dataset S2. A list of volatile organic compounds (VOCs) that were detected across all potato cultivar leaf samples.

Dataset S3. Differential volatile organic compounds in selected cultivars.

Dataset S4. Primer sequences used for gene cloning and qPCR validations.

Dataset S5. Primer sequences used for subcloning PopeORs and PopeOrco into the pGEMHE vector.

Dataset S6. List of volatile organic compounds (VOCs) used in electrophysiological (EAG) and TEVC assays.

Dataset S7. Primer sequences used for sgRNA synthesis and genotyping.

Dataset S8. Predicted secondary structure elements of PopeOR01, PopeOR15, and PopeOR73 proteins.

Dataset S9. Docking Results of PopeOR01, PopeOR15, and PopeOR73 with VOCs and binding data.
